# China's progress in synergetic governance of climate change and multiple environmental issues

**DOI:** 10.1093/pnasnexus/pgae351

**Published:** 2024-08-21

**Authors:** Jianxun Yang, Zhan Zhao, Wen Fang, Zongwei Ma, Miaomiao Liu, Jun Bi

**Affiliations:** State Key Laboratory of Pollution Control and Resource Reuse, School of the Environment, Nanjing University, Nanjing 210023, China; Institute for the Environment and Health, Nanjing University Suzhou Campus, Suzhou 215163, China; State Key Laboratory of Pollution Control and Resource Reuse, School of the Environment, Nanjing University, Nanjing 210023, China; Institute for the Environment and Health, Nanjing University Suzhou Campus, Suzhou 215163, China; State Key Laboratory of Pollution Control and Resource Reuse, School of the Environment, Nanjing University, Nanjing 210023, China; State Key Laboratory of Pollution Control and Resource Reuse, School of the Environment, Nanjing University, Nanjing 210023, China; State Key Laboratory of Pollution Control and Resource Reuse, School of the Environment, Nanjing University, Nanjing 210023, China; State Key Laboratory of Pollution Control and Resource Reuse, School of the Environment, Nanjing University, Nanjing 210023, China; Institute for the Environment and Health, Nanjing University Suzhou Campus, Suzhou 215163, China

**Keywords:** synergetic governance, carbon mitigation, environmental crisis, China

## Abstract

Advancing the synergetic control of climate change and environmental crisis is crucial for achieving global sustainable development goals. This study evaluates synergetic governance levels over climate change and four environmental issues at the provincial level in China from 2009 to 2020. Our findings reveal significant progress in China's coordinated efforts to mitigate carbon emissions, reduce air pollutants, and conserve water resources. However, there remains room for improvement in managing solid waste and protecting ecological systems and overall progress in synergetic governance has slowed since 2015. Employing a random forest model, we identify socio-economic factors with great influence on synergetic climate change and environmental governance, such as energy intensity, service sector development, electronic equipment manufacturing, and transportation. Additionally, we reveal nonlinear relationships between some factors and performance of environmental subsystems, including both plateau effects (e.g. output in the smelting of ferrous metals) and *U*-shaped patterns (e.g. output in the manufacturing of metal products), possibly attributed to constraints in end-of-pipe treatment capacities and complexities in supply chain networks. Furthermore, through hierarchical clustering analysis, we classify provinces into four groups and provide tailored recommendations for policymakers to enhance synergetic governance levels in their respective regions. The framework established in this study also serves as a valuable reference for countries seeking to develop practical and context-specific solutions to mitigate climate and environmental risks.

**Significance Statement**

This study assesses the coordinated management of climate change and four environmental issues across Chinese provinces from 2009 to 2020. Our findings reveal notable progress in China's endeavors to mitigate carbon emissions, reduce air pollutants, and conserve water resources. However, there are notable deficiencies in addressing solid waste management and ecological conservation, particularly evident after 2015. Utilizing a random forest model, we uncover nonlinear relationships between various socio-economic factors and climate and environmental governance. Furthermore, we identify pivotal drivers that hold promise for yielding benefits across multiple sectors. Tailored recommendations for enhancing the level of coordinated governance are provided for provinces categorized into distinct groups by hierarchical clustering analysis.

## Introduction

Climate change is considered one of the greatest threats to sustainable development, having unprecedented repercussions on both nature and human society (1, 2). More than 70 economies, including the biggest emitters like China, the United States, and the European Union have initiated ambitious net-zero targets to cut greenhouse emissions (3). However, climate change is only one of many current dangerous environmental problems. Many regions in the world are facing a series of different environmental issues. For instance, air pollution is responsible for a number of respiratory and heart diseases (4, 5), causing millions of premature deaths annually in populous developing economies like India and China (6, 7). One-quarter of the world's population, most of which are living in Middle East, North Africa, and South Asia, are exposed to extremely high levels of water scarcity and water pollution (8, 9), which threatens the countries' economic growth and food security (9, 10). Agriculture expansion and illegal logging drive alarmingly high deforestation in Brazil, India, and Indonesia, directly leading to local biodiversity loss and destruction of carbon sinks (11). Achieving 2030 Agenda for Sustainable Development requires tackling these critical environmental issues together, as many of them are closely related to certain specific sustainable development goals, targets, or indicators.

In local environmental regulation practices, climate change and environmental issues are always framed as isolated issues, suggesting that they could be solved in isolation (12). This is problematic because narrowly focused actions to tackle climate change or environmental issues are not enough to resolve them all. There are three primary reasons that decision-makers should consider synergy solutions to addressing these issues. Firstly, climate change and most environmental pollution are directly driven by intensive human economic activities and predominantly originated from similar sources such as the combustion of fossil fuels (13, 14). Accordingly, reducing greenhouse gas emissions and implementing environmental regulation calls for attention to increasing cost-effectiveness (15, 16). Secondly, the compounding impacts of environmental crisis and climate change are becoming intricately intertwined and reinforced each other (17, 18). A body of evidence indicates that climate hazards are likely to intersect with local pollution and ecological degradation (1, 19), exacerbating public health risks and unfolding economic crisis (20). Therefore, solutions to mitigate climate change and environmental risks simultaneously are urgently needed to ensure personal safety and protect public property. Thirdly, synergetic governance of climate change and other environmental issues may bring multidimensional co-benefits compared with individual system governance. For example, co-control of climate change and air pollution can help reduce the exposure to extreme temperature and heavy pollution compound events, resulting in significant health co-benefits. Pursuing low-carbon, high-efficiency resource use and pollutant emission reductions may also drive the lightening of industrial structures and cleaner energy, contributing to high-quality economic development.

In view of the significance to address climate change and multiple environmental crisis collectively, previous studies have evaluated country or city-level progresses on the synergetic control level of these challenges or propose potential synergetic roadmaps, but dominantly focused on carbon and air pollutants like particulate matters (PM_2.5_), ozone (O_3_), and sulfur dioxide (SO_2_) (21–23). Carbon mitigation and air pollution control can lead to remarkable co-benefits as these two share similar emitting sources and are linked in physical mechanisms (24–26). For example, a great number of scholars have assessed the effectiveness of synergetic control for CO_2_ and air pollutant in China, suggesting narrowed room for end-of-pipe mitigation but high potential for carbon-negative technologies in future synergetic roadmaps (25). Air quality-related health burden reduction is considered an important co-benefit of decarbonization policies in the United States, and modeling studies have provided optimized synergetic pathways to minimize regional and ethnical disparities (27). However, excessive attention on carbon and air pollutant synergy may hinder a systematic understanding of the complex interlinkages between climate actions and other environmental issues. Evidence shows that there hide potential tradeoffs between deployment of certain climate change mitigation measures and solutions to other environmental agenda (28). The booming expansion of renewable energy such as wind, solar, and bioenergy, for example, may cause environmental risks by changing land use patterns and damaging ecosystem functions, exacerbating regional water scarcity, and creating end-of-life solar panel and turbine waste (29–31). To advance multiple targets of the SDGs, it is informative to investigate the synergies between actions to address climate actions and control a broader list of environmental challenges.

Multiple social and economic factors may affect synergetic and tradeoff relationship between climate actions and the governance of other environmental issues (32). Identifying key driving factors and increasing their regulation priority can effectively enhance the level of synergetic management. Previous studies heavily rely on decoupling models and spatial econometric models to reveal factors that may constrain or promote the synergy level of pollution and carbon reduction (33). Their results generally suggest that cleaner industrial and energy consumption structure are associated with higher synergetic levels. However, there are several research gaps that weaken the practical significance of these findings. Firstly, these studies largely simplified complex nature–society interdependencies and dynamics, overlooking the nonlinear relationships between socio-economic and ecological systems (34). Policy targeted in one objective may counteract or reinforce the other through direct or indirect interlinkages across sectors (35). Ignoring complex nature of such nexus, climate actions might end up being counterproductive for the achievement of other environmental protection objectives and sustainability agendas. Secondly, environmental targets and driving factors considered in existing studies are limited. Many factors, such as the structure of refined manufacturing sectors and government financial expenditure, which may have indirect correlations with low-carbon initiatives and environmental performance, are frequently absent from these analyses (36–39). Finally, it is important to acknowledge the disparities in economic development levels and natural resources across the region, which consequently gives rise to varying environmental challenges. More actionable and regional tailored policy options are required for local decision-makers to develop priorities and achieve more efficient allocation of financial resources (40).

This study aims to fill the research gaps by introducing an integrated framework which assesses synergetic level of climate change and multiple environmental management objectives. China is the largest carbon emitter and the top polluted countries in the world. The Chinese government has redoubled efforts and released the stringent policies to curb carbon and pollution emission in the past decade. Yet, there still lacks systematic analysis of if climate change and other environmental objectives are progressed collectively.

Taking China as the case, we devised an index framework and deployed the entropy weight method to calculate the absolute governance scores of climate change and four types of environmental issues across 31 provinces in China from 2009 to 2020. We then applied the coupling coordination degree (CCD) model to evaluate their synergetic levels between climate actions and environmental regulations. The random forest (RF) algorithm was applied to model the nonlinear interlinkages between 19 socio-economic drivers and the performance of climate change environmental governance performance. Finally, the CCD scores were incorporated into the hierarchical cluster analysis to realize the classification of provinces. Targeted solutions for improving synergetic control of carbon and environmental systems were offered for provinces on a cluster basis.

The insights and lessons learned from the period spanning 2009 to 2020 are essential for China to address climate change and environmental challenges in the coming years. Other nations and regions can draw valuable information from this study devise practical and context-specific solutions. This approach aids in mitigating climate and environmental risks, fostering sustainable development on both a national and global scale.

## Results

### Absolute and synergetic governance levels of five subsystems

**Fig. 1.**
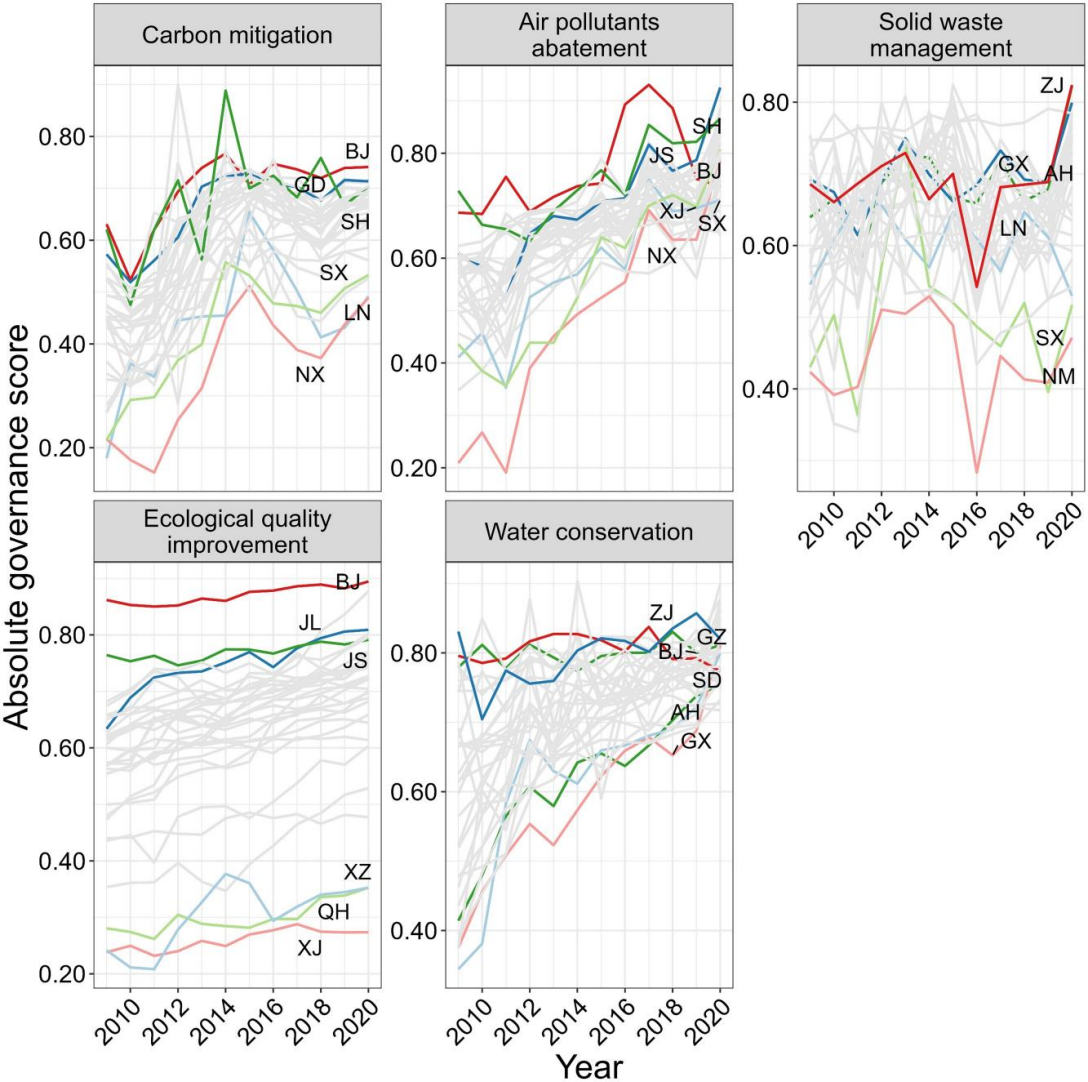
Absolute governance scores of climate change and environmental subsystems in 31 provinces. The color-coded lines represent the performance scores of the top three and bottom three provinces over time.

The interannual trend of the absolute governance score for each subsystem is depicted in Fig. 1. Efforts aimed at climate mitigation, air pollution abatement, and water resource conservation has proven effective across most provinces. The average absolute governance scores have shown substantial improvement from 2009 to 2020, with increases of over 30%. Specifically, the scores rose from 0.434 to 0.651 for climate mitigation, from 0.544 to 0.779 for air pollution abatement, and from their initial values to 0.797 for water resource conservation, as shown in Table S3. The score for climate change subsystem exhibited rapid growth until 2015, followed by a notable slowdown, showing a deceleration in carbon mitigation efforts post-2015. Furthermore, the scores for the solid waste subsystem and carbon mitigation subsystem showed significant fluctuations, particularly in provinces with high-energy extraction and production activities like Shanxi (SX) and Ningxia (NX), indicating the severity of their solid waste pollution and climate change. Figure S1 shows the interannual variability in the indicators contained by the carbon mitigation subsystem and the solid waste management. We found that the growth rate of carbon emissions and solid waste production of each province showed a fluctuating trend, while the carbon emission intensity, solid waste emission intensity, and per capita domestic waste removal volume changed slowly. Because the growth rate of carbon emissions and the growth rate of solid waste production occupy a large weight in the two subsystems (0.56 and 0.38, respectively), therefore, its fluctuation is the main reason why the absolute governance scores of the two subsystems show a significant upward or downward trend. The ecological system score displayed considerable provincial variations, primarily due to inherent differences in natural endowments across regions.

**Fig. 2.**
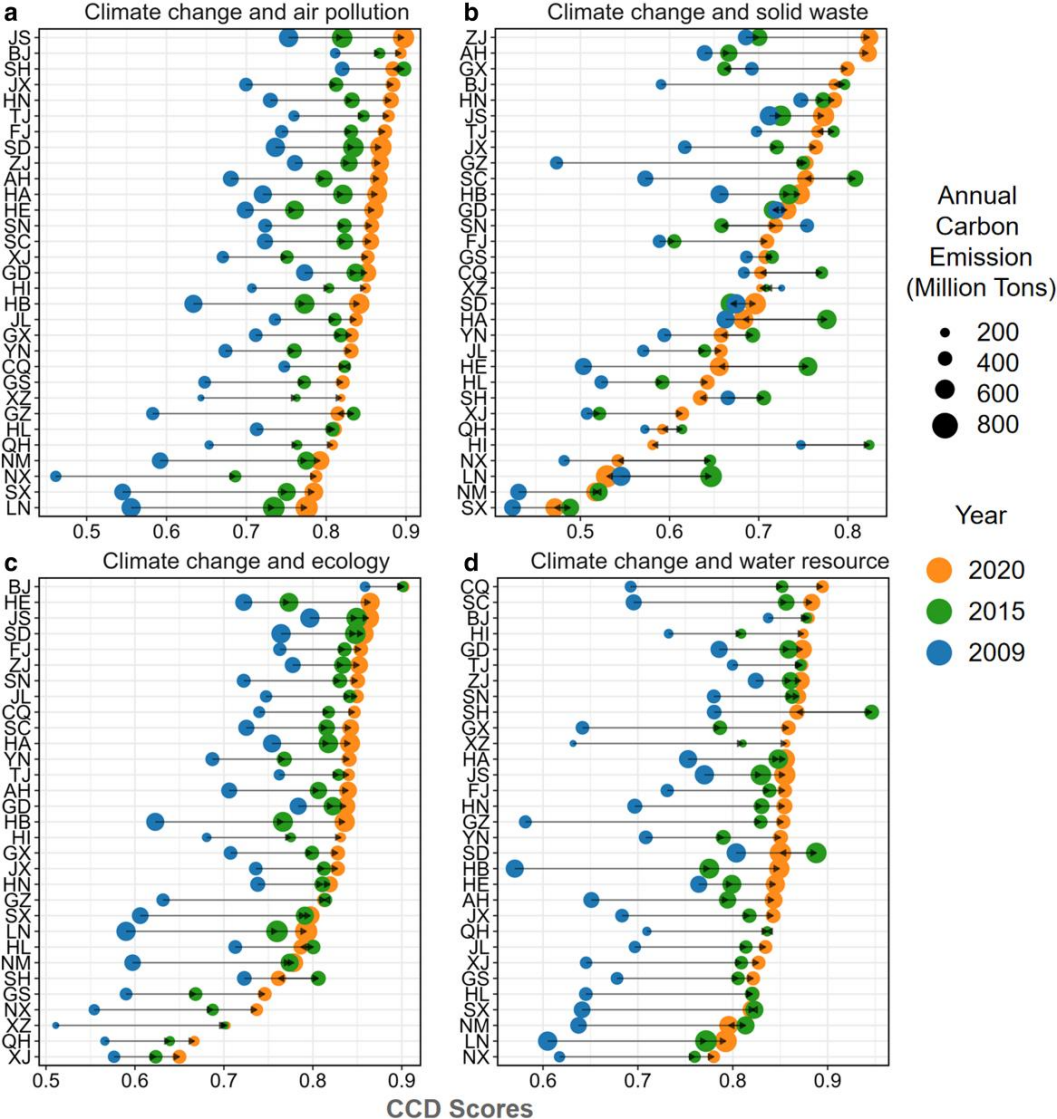
CCD scores of climate change and four environmental issues in 31 provinces. a–d) The CCD scores for carbon mitigation subsystem and air pollutants abatement, solid waste management, ecological quality improvement and water resources conservation subsystems. Dot colors denote different years. The size of the dot is proportional to the total carbon emission in 2020. Provinces are ranked according to their CCD scores in 2020.

Figure S2 shows the annual trend of CCD scores across various provinces. For a clearer depiction of the changes in the synergetic level for each province, Fig. 2 presents the CCD scores for the years 2009, 2015, and 2020, respectively. In the first half of the 2010s, progress in synergetic level improvement is more prominent than in the second half, aligning with the deceleration of carbon mitigation efforts after 2015. By 2020, China's carbon mitigation has shown strong synergies with air pollutants abatement and water resources conservation, with 27 and 28 provinces respectively achieving robust levels (CCD > 0.8). In contrast, the synergies between carbon mitigation and solid waste management and ecological quality are weaker, with more than 10 provinces still not achieving good coupling. In addition, in recent years, the contradiction between climate action and solid waste management in China has become more and more severe, with the CCD scores of the two subsystems in more than half of the provinces showing a downward trend.

Regarding specific provinces, municipalities such as Beijing (BJ) and Shanghai (SH) and coastal developed provinces such as Jiangsu (JS), Zhejiang (ZJ), and Fujian (FJ) have reached relatively higher levels of synergetic governance in addressing climate change and other environmental concerns by 2020. These provinces have high-emission density and population density but serve as exemplars of effective coordination and collaboration in carbon mitigation and environmental management. Conversely, provinces located in North and Western inland China have not achieved good coupling. These regions, including coal production-based energy provinces such as Shanxi (SX), Liaoning (LN), and Inner Mongolia (NM) face greater challenges in low-carbon energy transition and synergetic control of environmental issues due to carbon lock-in effects. Economically disadvantaged western provinces with limited natural resources, such as Ningxia (NX), Gansu (GS), and Qinghai (QH) have also shown lower performance in this regard. Breaking through the constraints imposed by natural resource endowments and achieving high-quality economic growth with carbon-pollution balance are the primary challenges faced by these regions.

### Driving factor analysis for the performance of each subsystem

**Fig. 3.**
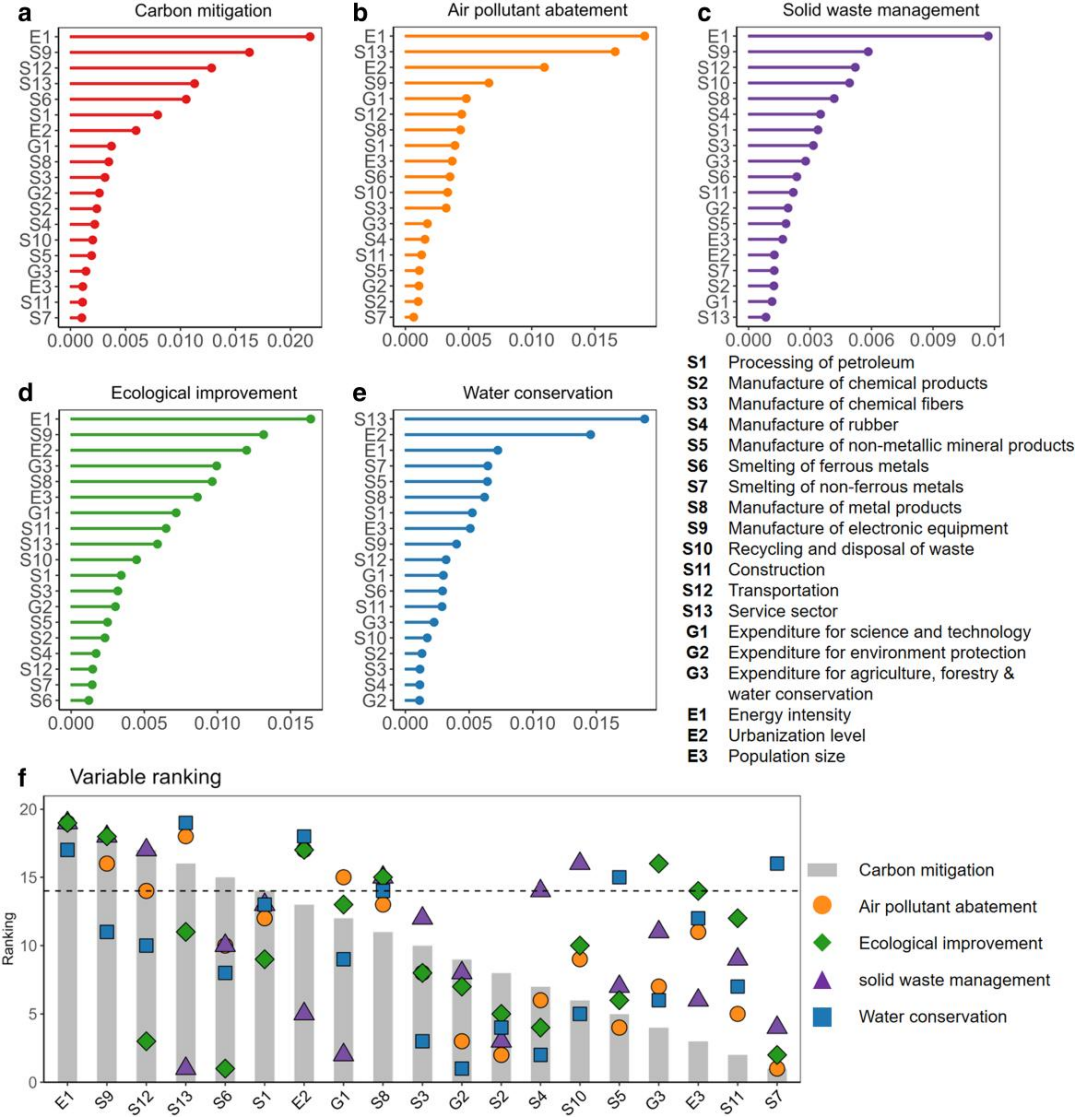
Significance of socio-economic predictors for each subsystem. a–e) The SHAP values of each variable in predicting the governance performance for each subsystem are depicted. f) The ranks of feature importance i.e. SHAP values for some top predictors across all subsystems are illustrated. The predictors were chosen by identifying the top six most significant variables within each subsystem, and their performance was compared across other subsystems. A larger value of ranking indicates a higher feature importance ranking with a greater influence on the subsystem. Ranks are indicated by gray bars for carbon system and by different shapes for environmental systems. A dotted line is added to aid in identifying their positions.

We utilized 19 socio-economic variables to build RF models aimed at predicting the absolute governance scores of five subsystems from 2009 to 2020. Employing 10-fold cross-validation, we evaluated the interpretability and accuracy of the model, and compared the evaluation results with those of fixed effects models. Figure S3 and Table S5 show that the interpretability and accuracy of the RF model are significantly better than the traditional linear regression model. The significance of features to the model output is quantified by Shapely Additive Explanation Approach (SHAP) value for each subsystem and illustrated by order in Fig. 3a–e. To compare the synergetic influence of different variables, we extracted the top six important variables with the highest mean SHAP value from each subsystem. We compared their ranks of feature importance across all subsystems, as shown in Fig. 3f.

The findings identify several factors that have a major impact on both carbon mitigation and multiple environmental subsystems. Among the top six factors influencing the carbon subsystem, energy intensity (E1), service sector (S13), sectoral outputs of the processing of petroleum (S1), electronic equipment manufacturing (S9), and transportation (S12) show high correlations with the performance of at least one other environmental subsystem. There are also predictors effective only in addressing multiple environmental problems but demonstrate less efficacy in carbon mitigation. For instance, sectors such as the manufacture of nonmetallic mineral products (S5), smelting of nonferrous metals (S7), the manufacture of metal products (S8), and recycling and disposal of waste (S10) are the most influential features only for water conservation, ecological protection and solid waste management, yet they exhibit less effectiveness in predicting carbon emissions. It sheds light on the pathways to promote integrated environmental management strategies and address carbon and other environmental issues simultaneously.

**Fig. 4.**
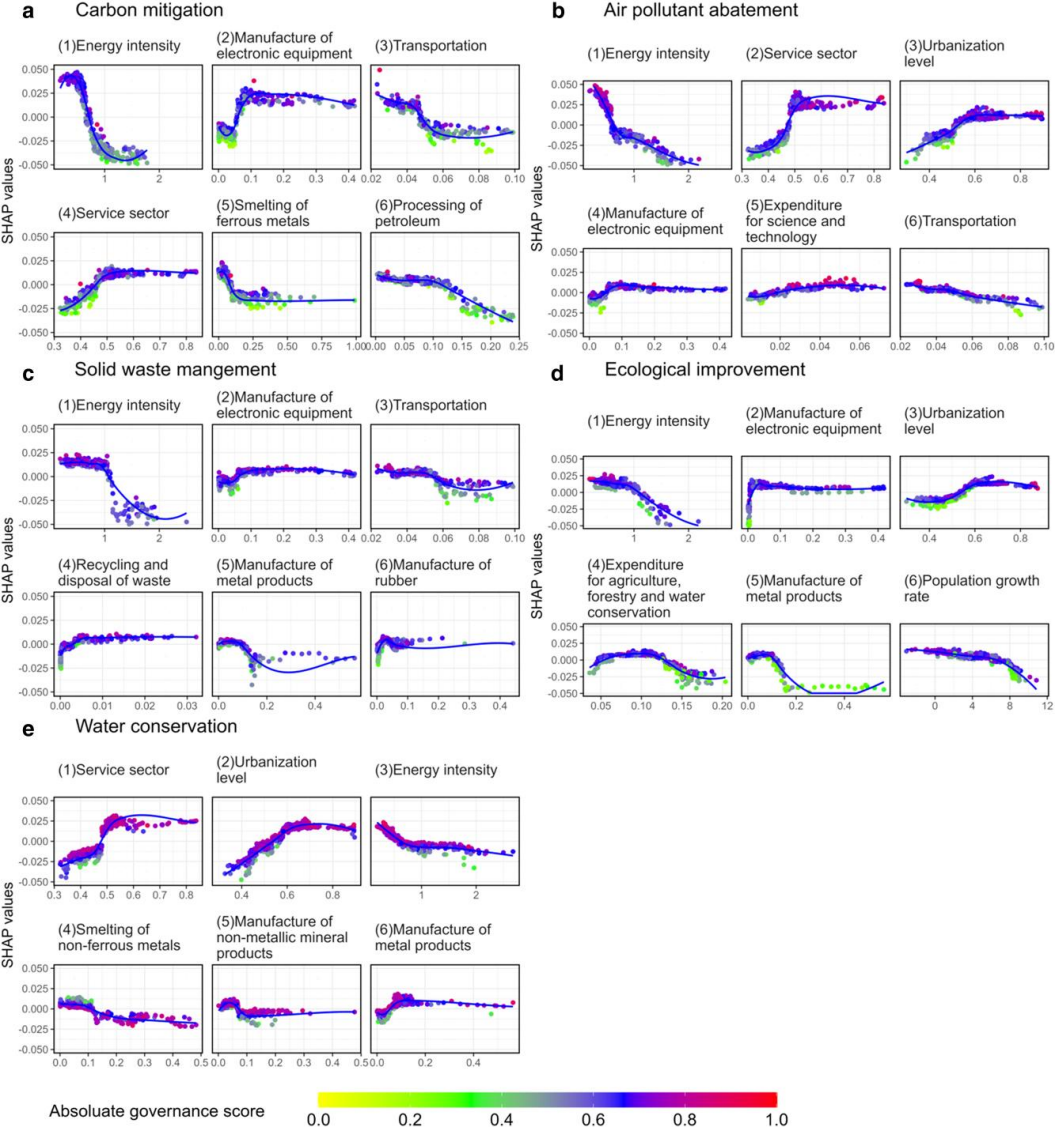
SHAP scatter dependence plots for each subsystem. a–e) The interaction between the top six driving factors and their SHAP values in predicting the governance performance of the corresponding subsystem is presented respectively. A locally weighted scatterplot smoothing line is added to illustrate the changing trend. The color of the dots indicates the absolute governance score of a certain subsystem of the province.

To investigate the direction in which predictors influence the performance of each subsystem, we drew scatter plots illustrating the relationship between the predictors and their corresponding SHAP values across 30 provinces (41). A positive SHAP value indicates that the variable positively influences the performance of the subsystem, while a negative value suggests the opposite. The curves for the top six predictors are presented in Fig. 4 for illustration and the rest can be found in Fig. S4. We observed highly nonlinear relationships between the variables and their SHAP values, proving the advantage of the RF-based model in capturing these complex correlations.

Among the five subsystems, the SHAP value of energy intensity (E1) all shows an overall decreasing trend with the growth of energy intensity, indicating that the developing model with high-energy-consumption hinders regional climate action and environmental governance. Therefore, reducing energy intensity remains one of the most crucial paths to improving environmental systems. Factors such as urbanization level (E2), the share of service industry (S13), expenditure for science and technology (G1), and the outputs of electronic equipment manufacturing industry (S9), which are related to the level of regional development, play a crucial role in advancing the governance of regional climate change and environmental issues. However, as these factors increase, there exists a plateau in enhancing governance levels. In other words, once reaching a certain level, solely relying on the adjustment of industrial structures may not drive further progress, necessitating the exploration of other emission reduction potentials.

Furthermore, the manufacturing industry exerts influence on all five subsystems, while exhibiting distinct characteristics within each. For instance, the smelting of ferrous metals (S6), including the production of steel and iron, initially diminishes the performance of the carbon subsystem. However, this diminishing trend weakens as the sector's volume expands. This might be owing to the strict emission standards implemented in China over the past decades, which have limited the environmental impacts of some major steel and iron-producing provinces.

The manufacture of metal products (S8), involving the fabrication, processing, and production of various metal-based goods, exhibits a relationship with SHAP values that has a reversed *U*-shape as the sector's output increases. Expansion of output within this sector at an early stage leads to improvements in solid waste management, ecological enhancement, and water conservation. However, this positive trend diminishes rapidly after surpassing a certain threshold. This observation may be attributed to the distinctive characteristics of the metal manufacturing sector, which spans various stages along the supply chain, from the upstream mining to downstream electronic equipment manufacturing. This diversity in the supply chain networks contribute to complex, nonlinear relationships between the sector and environmental pollution.

Finally, this study also focuses on considering the impact of population growth on regional environmental governance. Figure S4 shows the interactive relationship of the population growth rate with environmental issues. We found that the SHAP values of air pollutants abatement, ecological quality improvement, and water conservation subsystem all showed a significant decline with the increasing population growth rate, indicating that the rapid population growth hindered the solution of these environmental issues. The above findings confirm the conclusions of many related studies that the rapid population growth puts great pressure on air quality, ecological protection and residential water supply in local areas.

### Pathways to improve synergetic governance at the provincial level

**Fig. 5.**
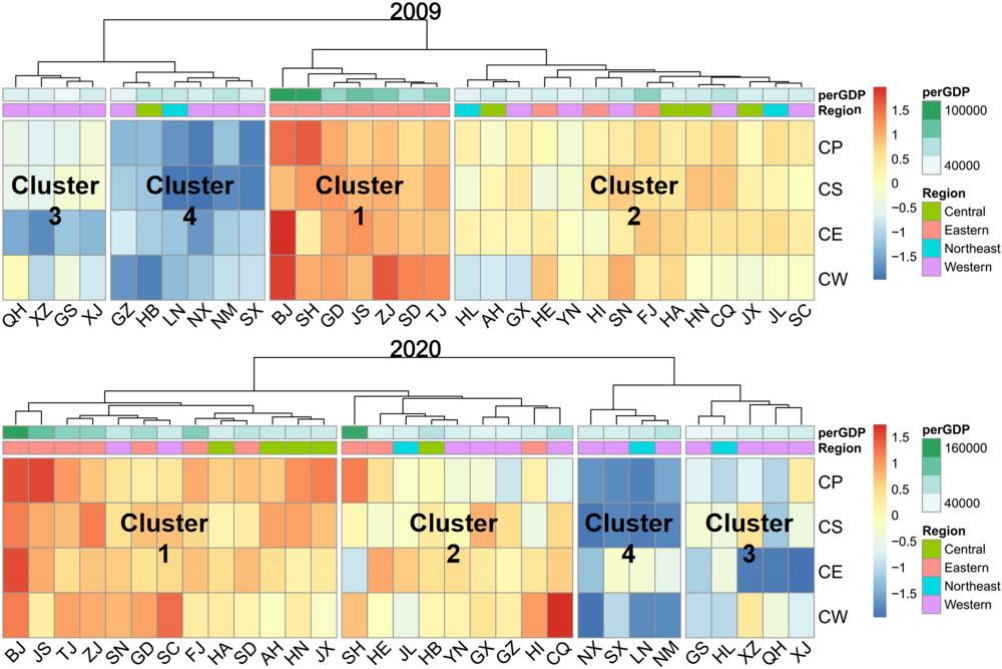
Provincial cluster analysis results according to the synergetic governance level in 2009 and 2020. CP, CS, CE, and CW indicate the CCD scores between carbon mitigation and air pollutants abatement, solid waste management, ecological quality improvement, and water conservation, respectively. The hierarchical clustering method identifies four groups of provinces with distinct features. The color of the square is proportional to standardized CCD scores, reflecting synergetic governance level of each province across climate change and other environmental subsystems. Per capita GDP and the geographical location of each province are added above the heat plot by different colors.

Taking the synergetic governance level of climate change and other environmental issues as variables, 31 provinces in China are divided into four categories by hierarchical clustering method for the year 2009 and 2020, respectively. The clustering results are shown in Fig. 5, and the characteristics of each cluster are detailed as follows.

Figure S6 shows the average synergistic governance levels across all provinces in the clusters. The four clusters display a declining trend in synergistic governance performance from high to low. Provinces in cluster 1 demonstrate the highest level of synergy between climate action governance and other environmental concerns. Cluster 2 provinces exhibit moderate levels of synergy, although some lag behind in effectively managing carbon and one other environmental subsystem. Cluster 3 provinces show even lower progress in synergistic governance levels, particularly in managing interactions between the carbon and ecological systems. Provinces in cluster 4 exhibit the least progress across almost all subsystems.

From 2009 to 2020, we observed a reshuffling of cluster memberships. Initially, cluster 1 comprised predominantly eastern provinces and municipalities with the highest per capita GDP across China. However, by 2020, an increasing number of central and western provinces had joined this group, indicating overall progress in environmental governance over the past decade. However, provinces in clusters 3 and 4, primarily situated in western China, continue to face low-synergy traps compared with their provinces. These regions either face economic disadvantages or serve as key energy-supply hubs in China and did not have very significant cluster changes during our study period.

Based on the clustering results and driving factor analysis outlined in previous sections, we then provide tailored recommendations for enhancing environmental synergy governance levels in the next phase. The provincial climate action in cluster 1 generally shows good synergy with the governance of other environmental problems, but some provinces are still restricted by some environmental subsystems. For instance, although Fujian (FJ) and Jiangxi (JX) have impressive carbon mitigation performance (ranked 4 and 12 in the provinces, respectively), their water management scores lag behind the other provinces (ranked 28 and 29 in the provinces, respectively), resulting in lower CCD scores for carbon-water subsystems. Figure S5 shows that the subsystems of each region are influenced by socio-economic variables. We find that improving the production processes of the nonmetallic mineral products industry and the nonferrous metal smelting industry and further promoting industrial transformation can be an effective strategy to improve the efficiency of water resources utilization, thereby enhancing synergetic governance levels of Fujian (FJ) and Jiangxi (JX).

For provinces in cluster 2, the level of synergetic governance for both climate change and other environmental subsystems is moderate. In 2020, In terms of synergetic governance between carbon-air pollution and carbon-solid waste systems, seven and five provinces in cluster 2 had lower CCD scores, respectively. These provinces have the potential to ascend to top tiers by exerting efforts on sectors that curb carbon emissions while simultaneously reducing emissions of air pollutants and solid waste. According to the analytical results of machine learning, provinces in cluster 2 should prioritize promoting industrial transformation, particularly by restraining the development of petroleum processing industry, fostering high-tech electronic industries, and advancing green transportation. These measures will effectively enhance the synergetic governance level in these provinces.

For provinces in cluster 3, there is a lack of coordination between climate action and other environmental issues, especially concerning the ecological system. The provinces in cluster 3 are primarily located in the northwest region and the Qinghai-Tibet Plateau (as shown in Fig. S7), such as Gansu (GS), Qinghai (QH), and Tibet (XZ). These regions exhibit low economic condition, relatively high carbon intensity, and extremely fragile ecology. Priority measures for these provinces should focus on initiatives that can improve ecological system performance and governance of other environmental issues simultaneously. Potential measures could include improving the production process of the metal products industry, increasing the level of urbanization and improving the construction of urban infrastructure. In addition, over-reliance on government investment in agriculture, forestry and water cannot effectively improve the level of environmental governance, so we should reasonably increase investment in science and technology and cultivate a number of high-tech industries such as electronic equipment manufacturing to reduce the damage of industrial production to the ecological environment.

Provinces in cluster 4 mainly comprise typical energy resource-based areas in China, rich in coal and metal, such as Shanxi (SX), Inner Mongolia (NM), and Liaoning (LN). The low performance regarding carbon-environment synergetic governance is primarily attributed to an economic development model heavily reliant on energy-consuming industries for energy production and manufacturing. Prioritizing improved performance in the carbon-reduction system is crucial for effectively increasing their synergetic governance levels. Potential pathways may include continually reducing energy intensity and gradually phasing out dependence on high-energy-consuming industries such as ferrous metal smelting and petroleum processing.

## Discussion

### Empirical evidence of policy effects

This study comprehensively assessed the performance of synergetic governance in climate change mitigation and four other environmental subsystems in China. During our study period, China has implemented some national environmental regulation projects, such as the Carbon Emissions Trading (CET) pilot (started in 2011), the Air Pollution Prevention and Control Action Plan (started in 2013), and the Water Pollution Prevention and Control Action Plan (started in 2015). It is estimated that billions of dollars have been invested in pollution mitigation. We employ fixed effect regression to explore the impact of these three policies on both the absolute governance level and the synergetic governance level of the environmental subsystems. The full analysis results are shown in Supporting Information.

Table S7 shows that the implementation of the Air Pollution Prevention and Control Action Plan has a significant positive impact on both the absolute governance level and the synergetic governance of carbon emission mitigation and air pollution control subsystems. Specifically, after the implementation of the policy, the provinces' absolute governance levels for air pollutants and carbon mitigation increased (*β* = 0.0613, *P* < 0.001; *β* = 0.1198, *P* < 0.001), and their synergetic governance level also rose (*β* = 0.0796, *P* < 0.001). This indicates that China's national air pollution control policy, initiated in 2013, and has promoted the coordinated control of climate change and air pollution.

Table S8 shows that the Water Pollution Prevention and Control Action Plan has also had significant and positive effects on the absolute governance and synergetic governance level of carbon mitigation and water resources management. After the implementation of the policy, the absolute governance levels for water conservation and carbon mitigation both increased (*β* = 0.0262, *P* < 0.05; *β* = 0.0482, *P* < 0.05), and their synergetic governance level also rose (*β* = 0.0265, *P* < 0.01). This suggests that the policy has improved inefficient water use and reduced carbon emissions to some extent.

Table S9 shows that the provinces that have introduced the CET Pilot perform better in carbon mitigation, air pollution abatement, and their synergies. However, the synergetic improvement in water conservation is not significant. Specifically, for the absolute governance levels and synergetic governance levels of the air pollution abatement and carbon mitigation subsystems, the improvements are evident (*β* = 0.1362, *P* < 0.05; *β* = 0.0959, *P* < 0.05; *β* = 0.0609, *P* < 0.05). For water conservation, the estimated coefficient of the carbon mitigation policy is not significant. These findings demonstrate that China's environmental policies have addressed the corresponding environmental issues and produced a certain degree of synergy. Yet, carbon mitigation policies are effective at simultaneously controlling carbon emissions and air pollution, but not for addressing other environmental issues.

### Policy implications

The study found that there are still gaps in China's current level of environmental governance compared with country-specific targets. Specifically, China has announced the goal to peak carbon emissions by 2030 and achieve net-zero emissions by 2060, yet there remains a significant gap regarding the carbon peak and neutrality targets. Additionally, China's current air quality standards largely fall behind the WHO recommended guidelines. To achieve the recommended air quality goals, it is estimated that a more than 90% reduction in air pollutant emissions compared with 2015 levels is needed, which remains challenging based on the progress observed in this study. China has also proposed ambitious “zero-waste city” goals, aimed at promoting more comprehensive utilization and lowering the environmental impacts of solid waste. However, our study clearly finds that the performance of solid waste management is not comparable to other sub-environmental systems and requires more effective policy support in the future.

Our findings have significant implications for designing more targeted policies to promote climate-environment synergetic governance. For major industrial areas, increasing process-integrated resource use efficiency and adopting end-of-pipe treatment techniques with synergetic control effects are effective pathways to achieve low-carbon development and address multiple environmental issues (42). It is essential to develop a comprehensive technical priority list for the synergetic control of climate and environmental issues. For example, promoting ultra-low emissions retrofitting in the steel, cement, coking industries, and boilers; enhancing energy-saving measures in wastewater treatment plants; advocating the use of sludge biogas cogeneration technology; and advancing the recycling of new types of waste such as decommissioned power batteries, photovoltaic modules, and wind turbine blades (43). Regions should consider their unique industrial realities and synergetic governance as highlighted in this study and promote the deployment of specific technologies or guide investment and R&D efforts toward these areas.

Secondly, industrial structure adjustment and spatial planning strategies should be established based on balancing carbon and multiple environmental issues. Specifically, energy and industrial development should be guided by regional environmental quality improvement goals (44). This includes the strategic planning of renewable energy projects, such as photovoltaic and wind power, on lands affected by abandoned mines, coal subsidence areas, closed landfills, and polluted sites (45). Strict entry policies should be enforced to prevent the indiscriminate development of high-energy-consuming, high-emission, and low-efficiency projects. A comprehensive approach to greening the entire industrial chain is required, accelerating source reduction, process control, end-of-pipe treatment, and comprehensive utilization within the industrial sector. Additionally, we find that certain sectors, such as electronic products, which are considered clean and high-tech industries, do not have a linear correlation with the level of environmental governance. This suggests that simply promoting high-tech industry development is not the sole solution for improving environmental performance. Therefore, regions should focus on upgrading traditional high-energy-consuming industries like chemical and steel toward more refined and greener practices, while also addressing the carbon-pollution synergies in emerging industries, such as data centers.

Thirdly, since climate and environmental governance involves a complex mix of policies, it is essential to enhance the synergy between these ecological, environmental, energy, and industry policies (46). Our findings indicate that carbon mitigation policies alone, while effective in mitigating carbon emissions, do not address multiple environmental issues. Therefore, it is necessary to ensure coordination and integration between different policies, including CET, electricity trading, dual control of energy consumption, and emission rights. For example, when defining the management boundaries and allocation schemes for CET, it is crucial to consider factors that promote both pollution reduction and carbon mitigation. Additionally, this study uses a relatively coarse scale at the provincial level. We recommend that related evaluations of pollution and carbon reduction synergies be conducted in key cities, industrial parks, and major enterprises. This will help guide regions in optimizing their coordinated management mechanisms and identifying issues across different scales.

### Contributions and limitations

This study contributes to related research areas in several ways. Firstly, we expanded the range of environmental elements included in the assessment of synergetic control levels. Beyond the carbon-air pollutant synergy typically considered in current literature, this study included indicators measuring synergetic governance between carbon mitigation and the management of solid waste, ecological condition, and water conservation. We identified provinces lagging behind in the management of some certain environmental factors, suggesting priorities for future improvement efforts. Next, we employed AI-based RF technique to analyze the driving factors of each environmental subsystem. This provided evidence of how certain factors may have feature importance in predicting single systems or multiple environmental systems together. It enabled us to build a large strategy pool for decision-makers to filter and identify specific strategies according to specific needs. Additionally, the RF model helped reveal nonlinear relationships between variables and identify distinct features that may explain more complex interactions between socio-economic drivers and environmental performance. Finally, we grouped provinces into four clusters using unsupervised machine learning techniques based on their performances in four types of synergetic climate-environment governance. This facilitated identification of clear pathways for provinces to improve their governance and achieve synergy across all environmental subsystems.

However, this study has a few limitations that need to be acknowledged. Data limitations constrained our ability to conduct longer time series analysis, and the indicator construction process for each system may not cover all aspects due to data constraints. Future research should include more systematic indicators and increase focus on key environmental issues such as soil erosion and wind and sand management. Additionally, we did not consider water quality as a sub-indicator of the water management system mainly due to the lack of long-term water quality monitoring data in China. Some methodological limitations may also require attention. For example, the CCD score method for synergetic analysis, while widely applied, may lose some validity when facing outliers or extreme values due to the min–max standardization process. Nevertheless, our conclusions remain robust, and the identified general patterns have implications for policymaking.

These limitations also point to potential research directions for future studies. Firstly, the definition of what constitutes good synergy remains controversial and deserves more discussions. In this study, the CCD score scheme emphasizes two main aspects of synergy. The first focuses on both systems reaching a highly favorable status, as measured by the development degree (T). The second highlights the synchronicity of change, as measured by the coupling degree (C), indicating that a unit change in one system is associated with improvements in other subsystems. The CCD score in this study treats these two types of synergy as equally important. However, we may anticipate more diverse definitions of synergy in future studies, with different weighting schemes when designing and combining these indicators. Also, we did not differentiate between the causal relationships between carbon and environmental performance in this study. For instance, whether high CCD scores indicate efforts to address climate change drive environmental improvements or vice versa.

Secondly, in this study, we only considered the synergetic governance of two systems and focused on interactions between the climate change and one other environmental system. Modeling without considering higher dimensions of synergetic governance may overlook complex interactions between environmental elements. For instance, certain industries, like those involving clean energy sources such as wind, solar, and lithium-ion batteries, can significantly enhance the synergetic control of carbon emissions and air pollution. However, the installation of these technologies may alter land use patterns, potentially causing ecological damage. Inadequate handling of waste generated by clean energy applications can lead to solid waste and water pollution. Therefore, focusing solely on the synergistic treatment of two aspects may disregard potential synergies or antagonistic effects on other environmental issues. In order to explore the synergy between climate action and comprehensive environmental governance in China, in the SI Appendix, the coupled coordination degree model is used to calculate the five-element synergetic governance scores of carbon mitigation in each province and other four environmental subsystems, and the obstacle degree model is used to diagnose the main environmental problems that restrict the improvement of the level of synergetic governance in different provinces (the construction method of the model is shown in the SI Appendix).

## Materials and methods

### Research framework

**Fig. 6.**
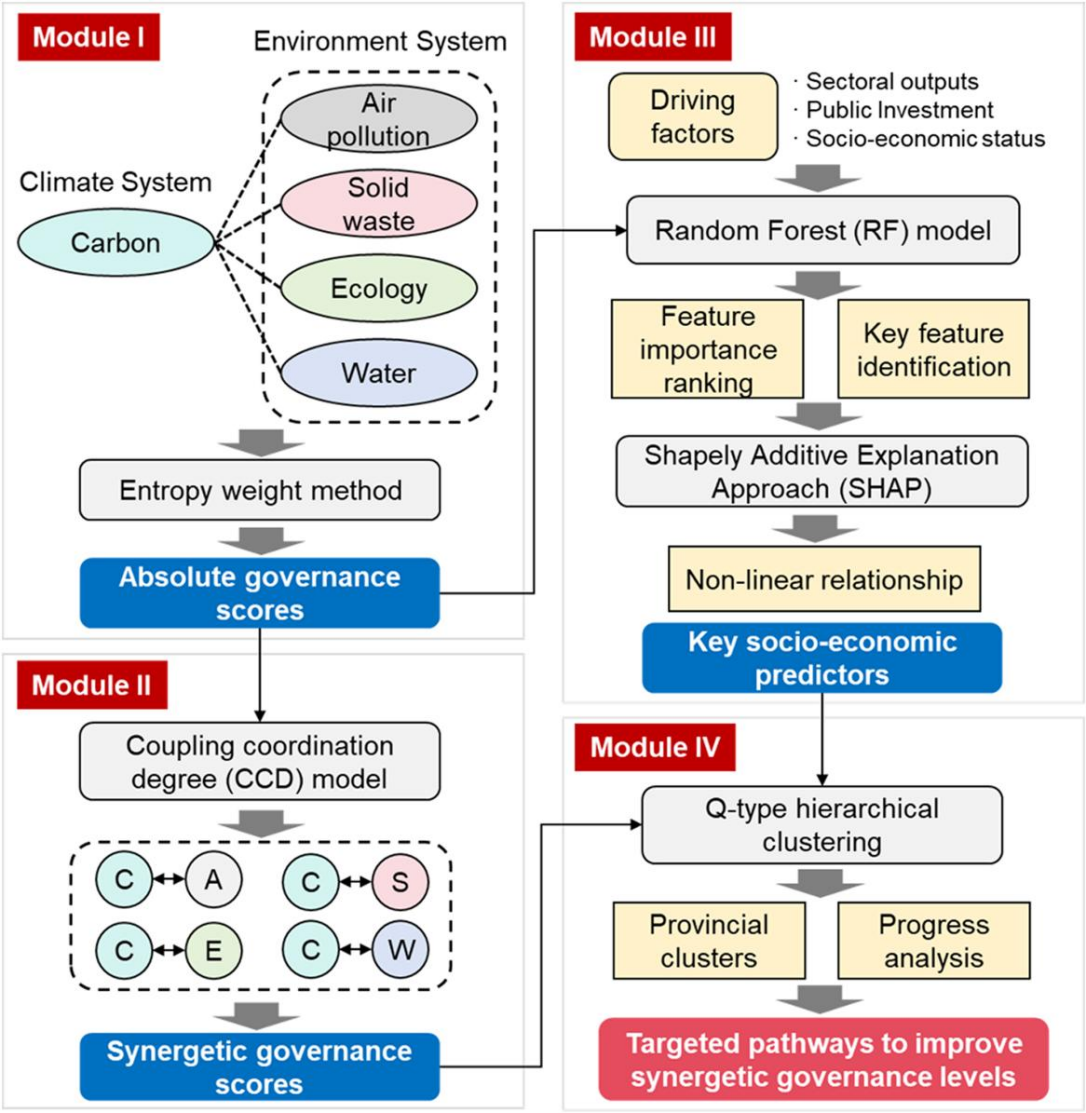
Research framework in this study.

To examine the synergetic governance of climate change and multiple environmental issues, we develop a research framework comprising four modules, as shown in Fig. 6 below.

In module I, an index framework was built to measure the governance performance of climate change and four types of environmental issues. Using the entropy weight method, we derived an absolute governance score for each subsystem. In module II, the absolute governance scores for the five systems are used to calculate the synergetic governance level. Specifically, we used the CCD model to calculate the degree of synergy for four pairs of absolute governance scores at the provincial level. In module III, the RF technique is employed to model the interaction between 19 socio-economic factors and the level of absolute environmental governance for each system. This helps identify the key socio-economic predictors of environmental performance. We also applied SHAP values to quantify the nonlinear relationships between these driving factors and the performance of environmental systems. Finally, in module IV, the provinces are classified and clustered according to the level of synergetic governance of climate change and environmental problems. The results from provincial clusters and key socio-economic predictors derived from module III are integrated to provide customized path measures for improving the level of synergetic governance. This study takes China as the case and detailed methods and data sources are reported in the following paragraphs.

### Measure the absolute governance level of each subsystem

We first built an index framework to measure the *absolute governance level* in five climate and environmental subsystems, including carbon mitigation, air pollutants abatement, solid waste management, water conservation, and ecological quality improvement, spanning from 2008 to 2020 for each province. The indicators used for each subsystem are detailed in Table 1.

**Table 1.** Indicators for each environmental subsystem.

Subsystems	Indicators	Type	Weights
Carbon mitigation	Growth rate of carbon emission	−	0.56
	Carbon emission intensity	−	0.44
Air pollutants abatement	Growth rate of SO_2_ emission	−	0.29
	SO_2_ emission intensity	−	0.18
	Growth rate of NO_X_ emissions	−	0.32
	NO_X_ emissions intensity	−	0.21
Solid waste management	Growth rate of solid waste emission	−	0.38
	Solid waste emission intensity	−	0.33
	Per capita domestic waste removal volume	−	0.29
Ecological quality improvement	Normalized Differential Vegetation Index	+	0.39
	Ecological water proportion	+	0.40
	Green coverage rate of built-up areas	+	0.21
Water conservation	Growth rate of water consumption	−	0.37
	Water consumption per unit GDP	−	0.15
	Water consumption per unit industrial output value	−	0.48

(+) represents a positive indicator, (−) represents a negative indicator.

China's current environmental strategy uses a dual approach, regulating both the total amount and intensity of pollutant emissions and resource use (47, 48). However, total emissions (or consumption) are closely tied to a region's economic size and population, making larger regions appears less effective in governance due to their higher emissions. To ensure fairness, this study evaluates subsystems—carbon mitigation, air quality improvement, water resource management, and solid waste management—using indicators for both the growth rate of total emissions (or consumption) and emission intensity (or consumption intensity). Those indicators were all collected at the province level.

More specifically, for the carbon mitigation subsystem, we considered indicators related to the annual change rate of total carbon emissions and carbon emission intensity (defined as the ratio of carbon emissions to GDP). The former reflects the absolute amount of carbon mitigation achieved in a given year, while the latter indicates the level of low-carbon economic activities.

For air pollution control, this study considers two main air pollutants: SO_2_ and nitrogen oxides (NOx), as they are prioritized in environmental regulations in China over the last few decades and have good statistical records. Annual change rate indicators are used to reflect the absolute emission reduction level in a given year, while intensity indicators are selected to reflect the extent to which economic activities have decoupled from air pollution emissions.

For solid waste management, we selected indicators representing both industrial and domestic sectors, as they are the primary sources of waste generation. The annual change rates of industrial solid waste and domestic waste are chosen to reflect their year-on-year reduction. The industrial solid waste per GDP indicator is used to assess the comprehensive utilization level of solid waste in industrial production. Per capita domestic waste removal volume index is then employed to reflect the pressure of residents on the solid waste management system.

For water resource conservation, we use indicators of annual change rate of total water consumption, industrial water consumption per unit of industrial added value, and total water consumption per unit of GDP. We use these two intensity indicators to account for regional differences in economic structure. Provinces where industrial sectors are dominant tend to have higher industrial water consumption per unit of industrial value added, while provinces that serve as major agricultural production hubs tend to have higher water consumption per unit of GDP (49).

We have selected three widely applied indicators to measure the health capacity of ecosystems in maintaining human well-being and protecting biodiversity (50). The normalized difference vegetation index quantifies the density and overall health status of terrestrial vegetation cover (33). The proportion of ecological water use reflects the amount of water resources that natural ecosystems can use to maintain their health and function. Urban green coverage indicates the extent of green space in urban areas, with higher coverage suggesting healthier ecosystems that provide benefits such as temperature regulation and improved air quality.

Considering the differences in dimensions and scales of the indicators, to ensure the comparability of evaluation results, this study used the extreme-value method (51) to standardize the raw data based on the Eqs. (1) and (2) to ensure the comparability of results (14):

Positive indicators:


(1) $$\begin{array}{c} Z_{ij}=\frac{X_{ij}-\min(X_{ij})}{\max(X_{ij})-\min(X_{ij})} \end{array}.$$

Negative indicators:


(2) $$\begin{array}{c} Z_{ij}=\frac{\max(X_{ij})-X_{ij}}{\max(X_{ij})-\min(X_{ij})} \end{array},$$

where, $X_{ij}$ and $Z_{ij}$ are the original value and standardized value of the *j*-th indicator of province *i*, respectively; and the normalized data interval is [0,1].

We then adopted the Entropy Weight Method to determine the weights according to the size of the indicator's variability (52). The calculation steps are presented by Eqs. (3) to (5):


(3) $$\begin{array}{c} \{\begin{array}{c} \;p_{ij}=\frac{z_{ij}}{\sum_{i=1}^{n}\;z_{ij}}\\ E_{j}=-\frac{1}{\mathrm{lnn}}\overset{n}{\underset{i=1}{\sum }}\;p_{ij}\ln p_{ij} \end{array} \end{array}$$

(4) $$\begin{array}{c} w_{j}=\frac{1-E_{j}}{\sum_{\;j=1}^{m}\;(1-E_{j})} \end{array}$$

(5) $$\begin{array}{c} U_{i}=\overset{m}{\underset{\;j=1}{\sum }}\;w_{j}\times z_{ij} \end{array}.$$

In these equations, *n* is the number of provinces and *m* is the number of indicators. $p_{ij}$ calculates the proportion of the index value of the province *i* under the indicator *j*. $E_{j}$ calculates the information entropy of indicator *j*. $w_{j}$ calculates the weight of indicator *j* (the results are shown in Table 1). Finally, this study uses the linear weighting method (53) to calculate $U_{i}$, the *absolute governance level* of each provincial subsystem.

### Synergetic level of addressing climate change and other environmental issues

We used CCD model to assess the level of synergetic governance across provinces in tackling climate change and other environmental concerns (54). The CCD indicator is composed of two parameters: coupling degree and development degree. The coupling degree reflects the extent of synergistic changes, or the degree of interdependence for the two subsystems in addressing environmental issues. The development degree indicates the quality or tangible progress of improvement achieved by the two subsystems. The specific equation is as follows:


(6) $$\begin{array}{c} C=\frac{2\times \sqrt[{2}]{\;f(U_{\mathrm{carbon}})\times f(U_{\mathrm{environment}})}}{\;f(U_{\mathrm{carbon}})+f(U_{\mathrm{environment}})} \end{array},$$

where *C* is the coupling degree, indicating the overall strength of the interaction between subsystems. $f(U_{\mathrm{carbon}})$ and $f(U_{\mathrm{environment}})$ are the *absolute governance level* of the climate change and environment subsystems. Next, we specify:


(7) $$\begin{array}{c} T=\alpha_{1}f(U_{\mathrm{carbon}})+\alpha_{2}f(U_{\mathrm{environment}}) \end{array}$$

(8) $$\begin{array}{c} \mathrm{CCD}=\sqrt{C\times T} \end{array}$$

*T* is the development degree, indicating the overall progress of absolute governance level for the two subsystem, which can reflect the quality of synergy. Then, $\alpha_{1}$ and $\alpha_{2}$ are the coefficients of weights to be referenced for the climate change and environment subsystems, respectively. In this study, we assumed that each subsystem is equally important to evaluate the development degree for each province, so $\alpha_{1}$ = $\alpha_{2}$ = 1/2. Finally, we calculated CCD scores to reflect the synergetic governance level for carbon emissions reduction and environmental governance. The higher the CCD values, the better performance of synergy achieved by the two subsystems.

### Al-based driver analysis of governance performance in each subsystem

We applied the RF model to explain drivers of *absolute governance level*, aimed to find practical solutions to promote the synergies of governance in multiple subsystems. In essence, environmental problems are generated by the complex interaction between human society and nature (55). Previous studies have reported a range of economic, industrial, and policy factors that may affect the performance of carbon and environmental systems. However, these factors are interconnected, and their influence on environmental performance is not linear. Traditional driver analysis techniques, such as regression and factor analysis, are susceptible to outliers when dealing with this type of problem, leading to a decrease in the predictive accuracy of the model (56). Moreover, these models assume that the features are independent of each other, and the results of the model may be inaccurate if there is multicollinearity between the features (57). Finally, these models can only express linear relationships and cannot handle complex nonlinear relationships (58). Therefore, we could adopt machine learning models because they are efficient in explaining nonlinear relationships and dealing with multicollinearity between variables, and they are not easily affected by outliers and have high prediction accuracy (59).

RF is a supervised machine learning algorithm widely used for both classification and regression tasks (60). It can handle high-dimensional data and does not necessarily require critical feature selection. This study explores the influences of 19 socio-economic factors on the levels of environmental governance, which vary greatly in their actual meanings. Machine learning models such as neural networks have high requirements for data preprocessing, and some variables may need to be deleted to improve the model's accuracy (61). In contrast, the RF model reduces the need for extensive data preprocessing and variable selection, making it a more flexible choice for this analysis (62). In addition, the RF model, being a tree-based model, exhibits high stability and is less prone to overfitting issues. Moreover, it executes data faster compared with boosting and other complex models (63).

We utilized a set of 19 distinct variables to train five RF models, each dedicated to one subsystem. Predictors considered in this study include urbanization level, population growth rate, share of tertiary industry, energy intensity, various industrial sub-sectoral outputs, and efforts for environmental regulations (see Table 2). Due to the lack of relevant data in Tibet (XZ), in order to ensure the availability of research data, we did not include it in the scope of the study.

**Table 2.** Socio-economic variables in random forest models.

Categories	Corresponding subindustry	Abbreviations	Calculation method
Industrial sectors	Processing of petroleum	S1	Output value/GDP
	Manufacture of chemical products	S2	Output value/GDP
	Manufacture of chemical fibers	S3	Output value/GDP
	Manufacture of rubber	S4	Output value/GDP
	Manufacture of nonmetallic mineral products	S5	Output value/GDP
	Smelting of ferrous metals	S6	Output value/GDP
	Smelting of nonferrous metals	S7	Output value/GDP
	Manufacture of metal products	S8	Output value/GDP
	Manufacture of electronic equipment	S9	Output value/GDP
	Recycling and disposal of waste	S10	Output value/GDP
	Construction	S11	Output value/GDP
	Transportation	S12	Output value/GDP
	Service sector	S13	Output value/GDP
Government investment	Expenditure for science and technology	G1	Expenditure/government fiscal expenditure
	Expenditure for environment protection	G2	Expenditure/government fiscal expenditure
	Expenditure for agriculture, forestry and water conservation	G3	Expenditure/government fiscal expenditure
Socio-economic development	Energy intensity	E1	Energy consumption/GDP
	Urbanization level	E2	Urban population/total resident population
	Population growth rate	E3	(Number of births per year − number of deaths per year)/Average annual population

Several critical parameters were considered during the model training process in this study. Firstly, by using the 10-fold cross-validation method, the original data set (comprising 360 samples from 30 provinces over 12 years) was randomly divided into 10 subsets of equal size. Nine subsets were sequentially merged as training sets, with the remaining subset used as the test set for each of the 10 training sessions. Secondly, we determined the number of trees to be included in the RF model. While a larger number of trees can enhance the stability and robustness of the model, it may also lead to increased computational time. In this study, we set the number of trees to 500, based on recommendations from previous studies (64). Thirdly, the maximum depth of trees which controls the level of granularity or complexity of the trees was set as 6. We then trained five separate RF models, one for each subsystem, using the predictors mentioned earlier. In addition, to show the advantage of the RF model over the traditional regression model, we trained the multiple linear regression models and the fixed effects regression model separately using the same predictors. We used root mean square error, coefficient of determination (*R*²), and mean absolute percentage error for each training session and averaged these values to assess and compare the model's interpretability and accuracy. The RF models were implemented using the “*randomForest*” package in R (version 4.3.0), and the traditional regression models were implemented using the “*lm*” package and “*plm*” package in R (version 4.3.0).

After building the RF models, we used the SHAP to assess the relative importance of each variable in predictions. SHAP calculates the marginal contribution of each feature to the model's output, assigning an importance score to each variable. Variables with higher absolute SHAP values are considered more important, given their greater impact on the model's predictions (65). The SHAP analysis in this study was performed relying on the “*treeshap*” package in R (version 4.3.0). We passed the five trained tree-based models to the functions and generated SHAP values for each feature in the dataset. We ranked the mean SHAP values of all features in each subsystem to find the most influential features driving specific predictions. SHAP scatter dependence plots were drawn to depict the interaction between driving factors and governance performance in each subsystem.

### Targeted pathways to improve synergetic governance levels

To provide suggestions for enhancing synergetic governance levels of climate change and environmental issues, we first used hierarchical clustering analysis, an unsupervised machine learning technique, to reduce dimensionality and classify all provinces. Hierarchical clustering does not require specifying the number of clusters beforehand, particularly useful when the optimal number of clusters is unknown. The CCD scores for synergetic governance of climate change and various environmental issues (including air pollution, solid waste, ecological systems, and water conservation) in 2009 and 2020 were used as inputs for the hierarchical clustering. The model was implemented based on the “*pheatmap*” package in R (version 4.3.0).

Next, based on the clustering results, we synthesized the main characteristics of each group and identified shortcomings in synergetic management of climate change and other environmental issues. We compared group memberships in 2009 and 2020 to assess the progress of synergetic governance levels over the past decade. This process supports the development of a final roadmap for provinces within each cluster to follow step-by-step.

Drawing upon the findings from the driver analysis, we then devised customized strategies for provinces in each group to enhance synergetic governance levels. This might involve promoting green management practices for specific socio-economic processes or expediting the industrial transformation process in sectors capable of yielding multiple environmental benefits. Ultimately, we offer a comprehensive strategy set that guides provinces toward achieving improved synergetic governance levels.

## Supplementary Material

pgae351_Supplementary_Data

## Data Availability

All study data are included in the article and SI Appendix.
