# Supplementary material for: China's progress in synergetic governance of climate change and multiple environmental issues
Source: PNAS Nexus. 2024 Aug 21;3(9):pgae351. doi: 10.1093/pnasnexus/pgae351 (PMC11370896; doi:10.1093/pnasnexus/pgae351)
Supplement: pgae351_Supplementary_Data [file pgae351_supplementary_data.docx]

**
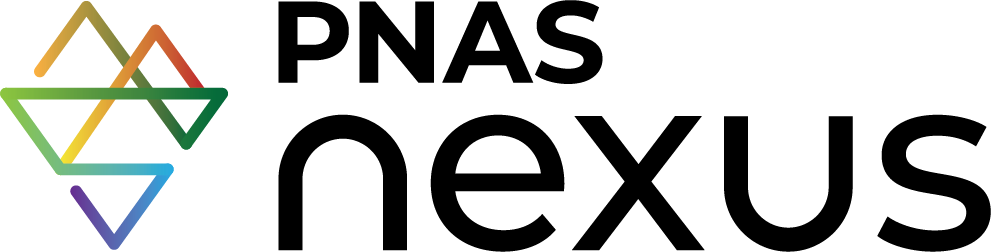
**

**Supplementary Information for**

**China’s progress in synergetic governance of climate change and multiple environmental issues**

Jianxun Yang^1,2,*^, Zhan Zhao^1,2^, Wen Fang^1^, Zongwei Ma^1^, Miaomiao Liu^1^, Jun Bi^1,2*^

^1^ State Key Laboratory of Pollution Control and Resource Reuse, School of the Environment, Nanjing University, China

^2^ Institute for the Environment and Health, Nanjing University Suzhou Campus, Suzhou 215163, China

*Corresponding authors. Jianxun Yang, Jun Bi

Email: yangjx@nju.edu.cn (Jianxun Yang); jbi@nju.edu.cn (Jun Bi)

**This PDF file includes:**

Supplementary Text

Figures S1 to S9

Tables S1 to S9

**Contents**

[**I. Data sources** 3](#_Toc173159614)

[**Table S1.** Overview of data involved 3](#_Toc173159615)

[**Table S2.** Abbreviations by provinces 4](#_Toc173159616)

[**II. Absolute and synergetic governance levels of five sub-systems** 5](#_Toc173159617)

[**Table S3.** Absolute governance level at the national level 5](#_Toc173159618)

[**Table S4.** Synergetic governance level at the national level 6](#_Toc173159619)

[**Figure S1.** The interannual variability in the indicators contained by the carbon mitigation subsystem and the solid waste management 7](#_Toc173159620)

[**Figure S2.** CCD score of climate change and four environmental issues in 31 provinces 8](#_Toc173159621)

[**III. Advantages of the random forest model over the traditional regression models** 9](#_Toc173159622)

[**Table S5.** Comparison of interpretability and accuracy of four models 10](#_Toc173159623)

[**Figure S3.** Comparison between predicted and actual values of four models 11](#_Toc173159624)

[**Table S6.** Results of the fixed effects model analysis of the importance of socio-economic factors 12](#_Toc173159625)

[**IV. Driving factor analysis for the performance of each subsystem** 13](#_Toc173159626)

[**Figure S4.** SHAP scatter dependence plots for each subsystem 14](#_Toc173159627)

[**Figure S5.** Significance of socio-economic predictors for each subsystem at the provincial level 17](#_Toc173159628)

[**V. Provinces are categorized according to the** **synergetic governance levels** 20](#_Toc173159629)

[**Figure S6.** Comparing the synergetic governance level among the different clusters 20](#_Toc173159630)

[**Figure S7.** Results of the spatial distribution of the clusters 21](#_Toc173159631)

[**VI. Impact of environmental policy implementation on synergistic governance** 22](#_Toc173159632)

[**Table S7.** The impact of air quality policy on the synergetic governance 22](#_Toc173159633)

[**Table S8.** The impact of water conservation policy on synergetic governance 23](#_Toc173159634)

[**Table S9.** The impact of carbon mitigation policy on synergetic governance 24](#_Toc173159635)

[**VII. Methodology for the construction of a higher dimensional synergistic governance model** 25](#_Toc173159636)

[**VIII. Analysis of the synergistic effect and obstacle degree between climate action and comprehensive environmental governance** 26](#_Toc173159637)

[**Figure S8.** The five-element synergetic governance scores of carbon mitigation in each province and other four environmental subsystems 27](#_Toc173159638)

[**Figure S9.** The results of the obstacle degree analysis of the subsystem 28](#_Toc173159639)

[**References** 29](#_Toc173159640)

I. Data sources

**Table S1.** Overview of data involved

| **Subsystems** | **Indicators** | **Data source** | **Unit** |
| --- | --- | --- | --- |
| Carbon mitigation | carbon emission | EDGAR（Emissions Database for Global  Atmospheric Research） | ton |
| Air pollutants abatement | SO_2_ emission | China Statistical Yearbook and Provincial  Statistical Yearbook | 10^4^ tons |
|  | NO_x_ emission |  |  |
| Solid waste management | solid waste emission  domestic waste removal volume | China Statistical Yearbook and Provincial  Statistical Yearbook | 10^4^ tons |
| Ecological quality improvement | Normalized Differential  Vegetation Index (NDVI) | NASA MOD13A3 | — |
|  | Ecological water consumption | China Environmental Statistics Yearbook | 10^8^ m^3^ |
|  | Green coverage rate of built-up areas | China Statistical Yearbook and Provincial  Statistical Yearbook | % |
| Water conservation | Water consumption | Provincial Water Resources Statistical Bulletin | 10^8^ m^3^ |
| Industrial sectors | Manufacturing sub-sector | China Industrial Statistics Yearbook | 10^4^ ￥ |
|  | Construction | China Statistical Yearbook and Provincial Statistical Yearbook | 10^4^ ￥ |
|  | Transportation |  |  |
|  | Service sector |  |  |
| Government investment | Expenditure for science and technology | China Statistical Yearbook and Provincial Statistical Yearbook | 10^4^ ￥ |
|  | Expenditure for environment protection |  |  |
|  | Expenditure for agriculture, forestry and water conservation |  |  |
| Socio-economic development | Energy consumption | China Energy Statistical Yearbook | tons of standard coal |
|  | Urbanization level | China Statistical Yearbook and Provincial Statistical Yearbook | % |
|  | GDP |  | 10^4^ ￥ |
|  | Population |  | 10^4^ persons |

**Table S2.** Abbreviations by provinces

| **Full name** | **Abbreviations** | **Full name** | **Abbreviations** | **Full name** | **Abbreviations** | **Full name** | **Abbreviations** |
| --- | --- | --- | --- | --- | --- | --- | --- |
| Anhui | AH | Hainan | HI | Jilin | JL | Sichuan | SC |
| Beijing | BJ | Hebei | HE | Liaoning | LN | Shanghai | SH |
| Chongqing | CQ | Henan | HA | Inner Mongolia | NM | Tianjin | TJ |
| Fujian | FJ | Heilongjiang | HL | Ningxia | NX | Tibet | XZ |
| Gansu | GS | Hubei | HB | Qinghai | QH | Xinjiang | XJ |
| Guangdong | GD | Hunan | HN | Shaanxi | SN | Yunnan | YN |
| Guangxi | GX | Jiangsu | JS | Shandong | SD | Zhejiang | ZJ |
| Guizhou | GZ | Jiangxi | JX | Shanxi | SX |  |  |

II. Absolute and synergetic governance levels of five sub-systems

Based on the absolute and synergistic governance scores of the five subsystems in the 31 provinces, we calculated the absolute and synergistic governance scores of the five subsystems at the national level by taking averages and analyzed their trends over time. Table.S3 shows the absolute governance scores of the five subsystems at the country level and their growth rates over the entire study period relative to the initial year. Table.S4 shows the synergistic governance scores of the five subsystems at the national level and their growth rates over the entire study period relative to the initial year. Fig. S1 shows the interannual variability in the indicators contained by the carbon mitigation subsystem and the solid waste management. Fig. S2 shows the trend of the scores of synergistic governance of the five subsystems at the provincial level over time.

|  | Absolute governance level | | | | |
| --- | --- | --- | --- | --- | --- |
| Year | Carbon mitigation | Air pollutants abatement | Solid waste management | Ecological quality improvement | Water conservation |
| 2009 | 0.43 | 0.54 | 0.62 | 0.56 | 0.60 |
| 2010 | 0.41 | 0.53 | 0.61 | 0.57 | 0.63 |
| 2011 | 0.45 | 0.54 | 0.61 | 0.58 | 0.66 |
| 2012 | 0.53 | 0.61 | 0.68 | 0.60 | 0.72 |
| 2013 | 0.59 | 0.63 | 0.68 | 0.61 | 0.70 |
| 2014 | 0.65 | 0.64 | 0.67 | 0.61 | 0.73 |
| 2015 | 0.67 | 0.68 | 0.69 | 0.62 | 0.72 |
| 2016 | 0.65 | 0.67 | 0.61 | 0.63 | 0.74 |
| 2017 | 0.63 | 0.75 | 0.64 | 0.65 | 0.75 |
| 2018 | 0.60 | 0.72 | 0.64 | 0.66 | 0.76 |
| 2019 | 0.64 | 0.70 | 0.63 | 0.67 | 0.77 |
| 2020 | 0.65 | 0.78 | 0.69 | 0.68 | 0.80 |
| Growth rate (from 2009 to 2020) | 49.99% | 43.18% | 11.29% | 22.39% | 33.89% |

**Table S3.** Absolute governance level at the national level

**Table S4.** Synergetic governance level at the national level

| Synergetic governance level | | | | |
| --- | --- | --- | --- | --- |
| Year | CP | CS | CE | CW |
| 2009 | 0.69 | 0.71 | 0.69 | 0.7 |
| 2010 | 0.68 | 0.71 | 0.69 | 0.71 |
| 2011 | 0.69 | 0.72 | 0.71 | 0.73 |
| 2012 | 0.75 | 0.77 | 0.75 | 0.78 |
| 2013 | 0.78 | 0.79 | 0.77 | 0.8 |
| 2014 | 0.8 | 0.81 | 0.79 | 0.83 |
| 2015 | 0.82 | 0.82 | 0.8 | 0.83 |
| 2016 | 0.81 | 0.79 | 0.8 | 0.83 |
| 2017 | 0.82 | 0.79 | 0.79 | 0.83 |
| 2018 | 0.81 | 0.78 | 0.79 | 0.82 |
| 2019 | 0.82 | 0.79 | 0.8 | 0.84 |
| 2020 | 0.84 | 0.82 | 0.81 | 0.85 |
| Growth rate (from 2009 to 2020) | 21.74% | 15.49% | 17.39% | 21.43% |

**Figure S1.** The interannual variability in the indicators contained by the carbon mitigation subsystem and the solid waste management


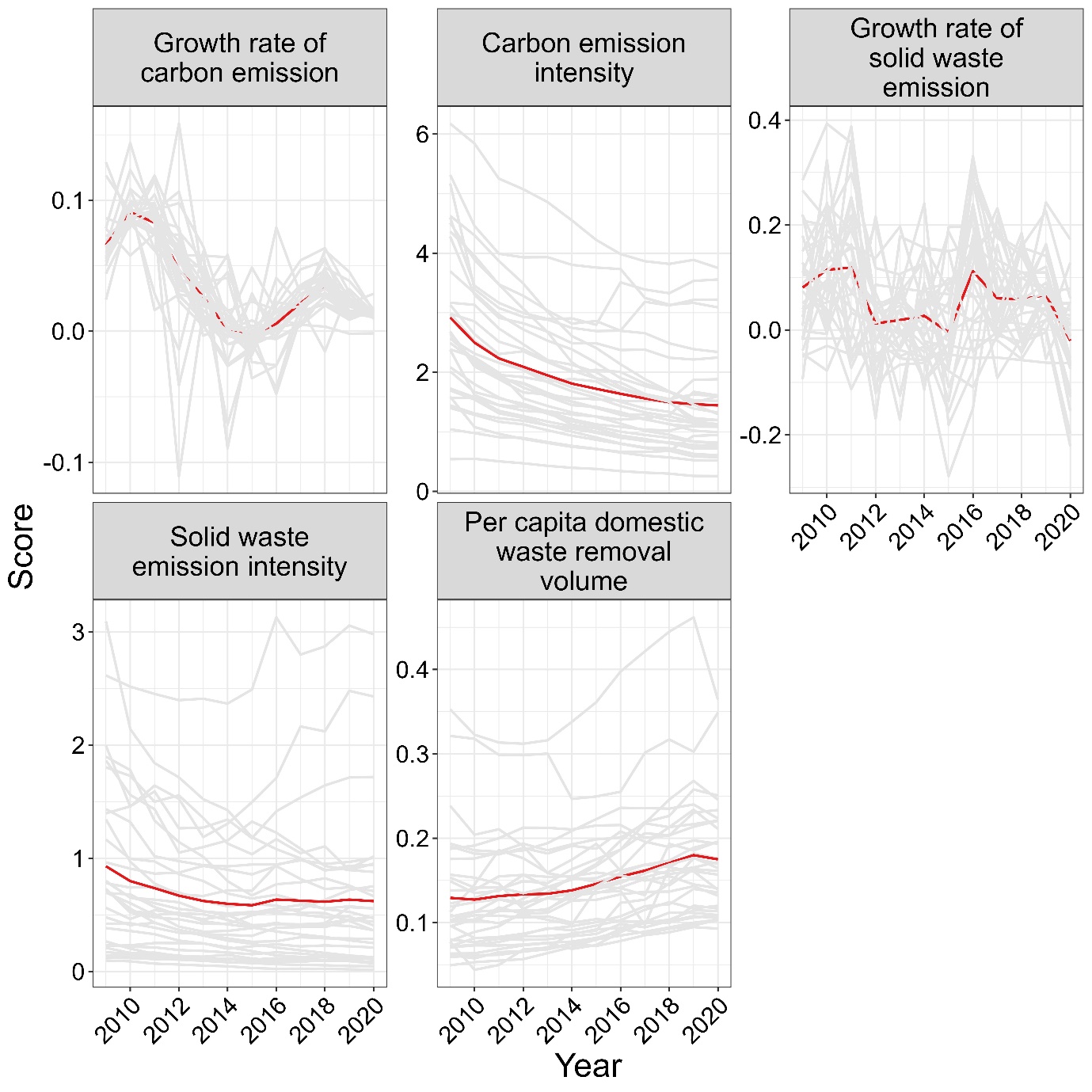


Note: We found that the growth rate of carbon emissions and solid waste production of each province showed a fluctuating trend, while the carbon emission intensity, solid waste emission intensity and per capita domestic waste removal volume changed slowly.

**Figure S2.** CCD score of climate change and four environmental issues in 31 provinces


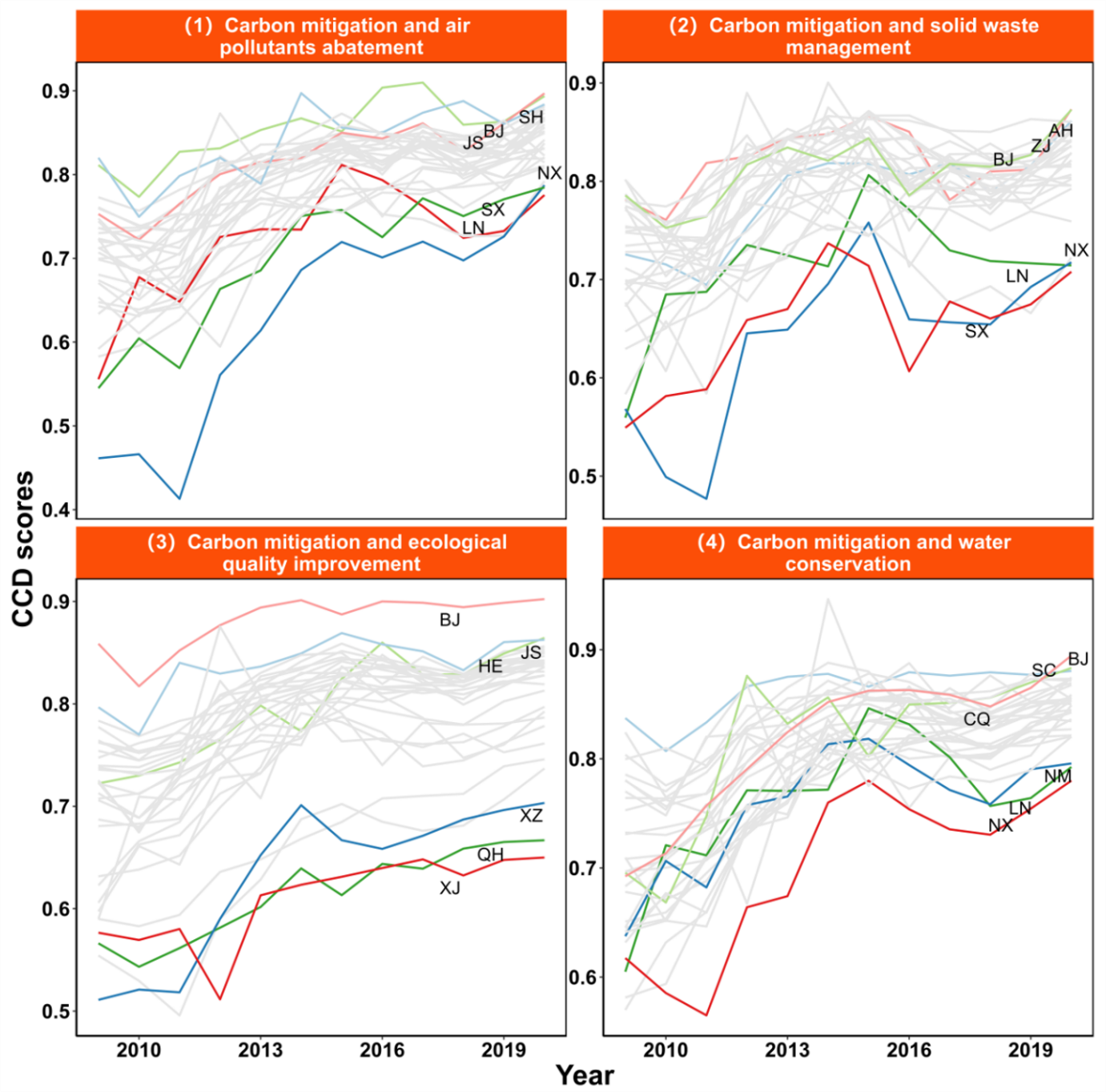


Note: We find that the synergy of carbon mitigation with air pollutants abatement and water conservation show a significant upward trend in most provinces, while the synergy with solid waste management fluctuated greatly. In addition, the synergistic effect of carbon mitigation and ecological protection was very different between different provinces, and the growth was slow during the study period. The color of the line highlights the change in the synergetic governance level of the top and bottom provinces over time. We found that provinces with low synergies between carbon mitigation and air pollutants abatement, solid waste management and water conservation have coal as the main energy source and are extremely dependent on the energy industry economically. However, provinces with weak synergies between carbon mitigation and ecological protection tend to be economically backward and have limited natural resources, such as Xinjiang (XJ) and Qinghai (QH).

III. Advantages of the random forest model over the traditional regression models

Using the 10-fold cross-validation method, we randomly divided the original data set into 10 subsets with the same number of samples. We then sequentially used 9 subsets as training sets and the remaining subset as the test set (1). The training set samples were used to train the following models: 1) Multiple Linear Regression (MLR); 2) Fixed Effects Regression controlling for individual effects (FE individual); 3) Fixed Effects Regression controlling for time effects (FE time); 4) Random Forest model (FR).

After training, the models were used to predict the scores of each subsystem for both the training and test sets, and these predictions were compared with the actual values. The comparison between the predicted and actual values for the four models is shown in Figure. S3.

Overall, we find that the deviation between the predicted and actual values is smallest for the random forest model, while it is largest for the multiple linear regression model. This indicates that the prediction accuracy of the random forest model is higher than that of the fixed effects regression model and the multiple linear regression model. Additionally, from the figure, we observe that the random forest model performs worse on the test set compared to the training set, indicating that the model has not been overfitted.

We also calculate the root mean square error (RMSE), coefficient of determination (R²), and mean absolute percentage error (MAPE) for each model to assess their interpretability and accuracy. Generally, models with lower RMSE and MAPE and higher R² values are considered to have a good regression effect (2). Table.S5 shows these performance indicators for each model. We found that the RMSE and MAPE of the random forest model for each subsystem were lower than those of the other two models, while the R² of the random forest model was significantly higher than that of the other two models. This indicates that the interpretability and accuracy of the random forest model are better than those of the multiple linear regression model and the fixed effects regression model.

Also, we compare the difference between the outputs of the random forest model and the traditional regression model. We set 19 socio-economic driving factors as independent variables and performed the fixed effect linear regression model to measure the effect and direction of their influence on the absolute governance level of the five environmental subsystems. The regression results are compared with the results performed by random forest model in this study. Table.S6 shows the regression coefficients of socio-economic variables in the five regression models.

We find that linear model cannot comprehensively identify the effect of socio-economic factors on environmental governance levels. In particular, the carbon emission reduction subsystem and solid waste management subsystem have omitted a large number of important influencing factors, such as metal smelting industry, electronic equipment manufacturing industry, transportation and service industry, etc. The results of this study show that there is a complex non-linear relationship between these industries and carbon emission reduction and solid waste management (see Figure. 4). However, the traditional fixed effect regression model cannot identify the importance of these variables.

In addition, the fixed effect regression results show that there is a negative correlation between the transportation and service sectors and the improvement of ecological quality, while the results of RF model show that they rise first and then decline. Therefore, we conclude RF are more advantageous in identifying these non-linear relationships which may help guide more accurate policy-making process.

**Table S5.** Comparison of interpretability and accuracy of four models

|  | Model | Dataset | Carbon mitigation | Air pollutants abatement | Solid waste management | Ecological quality improvement | Water conservation |
| --- | --- | --- | --- | --- | --- | --- | --- |
| MAPE | FE (individual) | train | 0.08 | 0.058 | 0.074 | 0.028 | 0.049 |
|  | FE (time) | train | 0.078 | 0.058 | 0.081 | 0.096 | 0.066 |
|  | MLR | train | 0.103 | 0.074 | 0.088 | 0.097 | 0.075 |
|  | RF | train | 0.039 | 0.03 | 0.036 | 0.022 | 0.026 |
|  | FE (individual) | test | 0.096 | 0.069 | 0.086 | 0.035 | 0.058 |
|  | FE (time) | test | 0.092 | 0.065 | 0.087 | 0.106 | 0.072 |
|  | MLR | test | 0.111 | 0.08 | 0.093 | 0.104 | 0.08 |
|  | RF | test | 0.092 | 0.058 | 0.086 | 0.051 | 0.05 |
| RMSE | FE (individual) | train | 0.057 | 0.048 | 0.06 | 0.022 | 0.045 |
|  | FE (time) | train | 0.053 | 0.047 | 0.064 | 0.076 | 0.061 |
|  | MLR | train | 0.071 | 0.06 | 0.071 | 0.077 | 0.071 |
|  | RF | train | 0.029 | 0.025 | 0.029 | 0.017 | 0.025 |
|  | FE (individual) | test | 0.067 | 0.058 | 0.07 | 0.066 | 0.054 |
|  | FE (time) | test | 0.058 | 0.052 | 0.07 | 0.083 | 0.067 |
|  | MLR | test | 0.076 | 0.065 | 0.075 | 0.08 | 0.075 |
|  | RF | test | 0.048 | 0.049 | 0.069 | 0.039 | 0.048 |
|  | FE (individual) | train | 0.804 | 0.803 | 0.569 | 0.974 | 0.784 |
|  | FE (time) | train | 0.831 | 0.812 | 0.507 | 0.687 | 0.603 |
| R^2^ | MLR | train | 0.688 | 0.689 | 0.404 | 0.681 | 0.469 |
|  | RF | train | 0.962 | 0.959 | 0.929 | 0.99 | 0.962 |
|  | FE (individual) | test | 0.722 | 0.725 | 0.43 | 0.965 | 0.690 |
|  | FE (time) | test | 0.799 | 0.772 | 0.433 | 0.622 | 0.516 |
|  | MLR | test | 0.658 | 0.645 | 0.35 | 0.645 | 0.399 |
|  | RF | test | 0.834 | 0.796 | 0.475 | 0.958 | 0.749 |

**Figure S3.** Comparison between predicted and actual values of four models


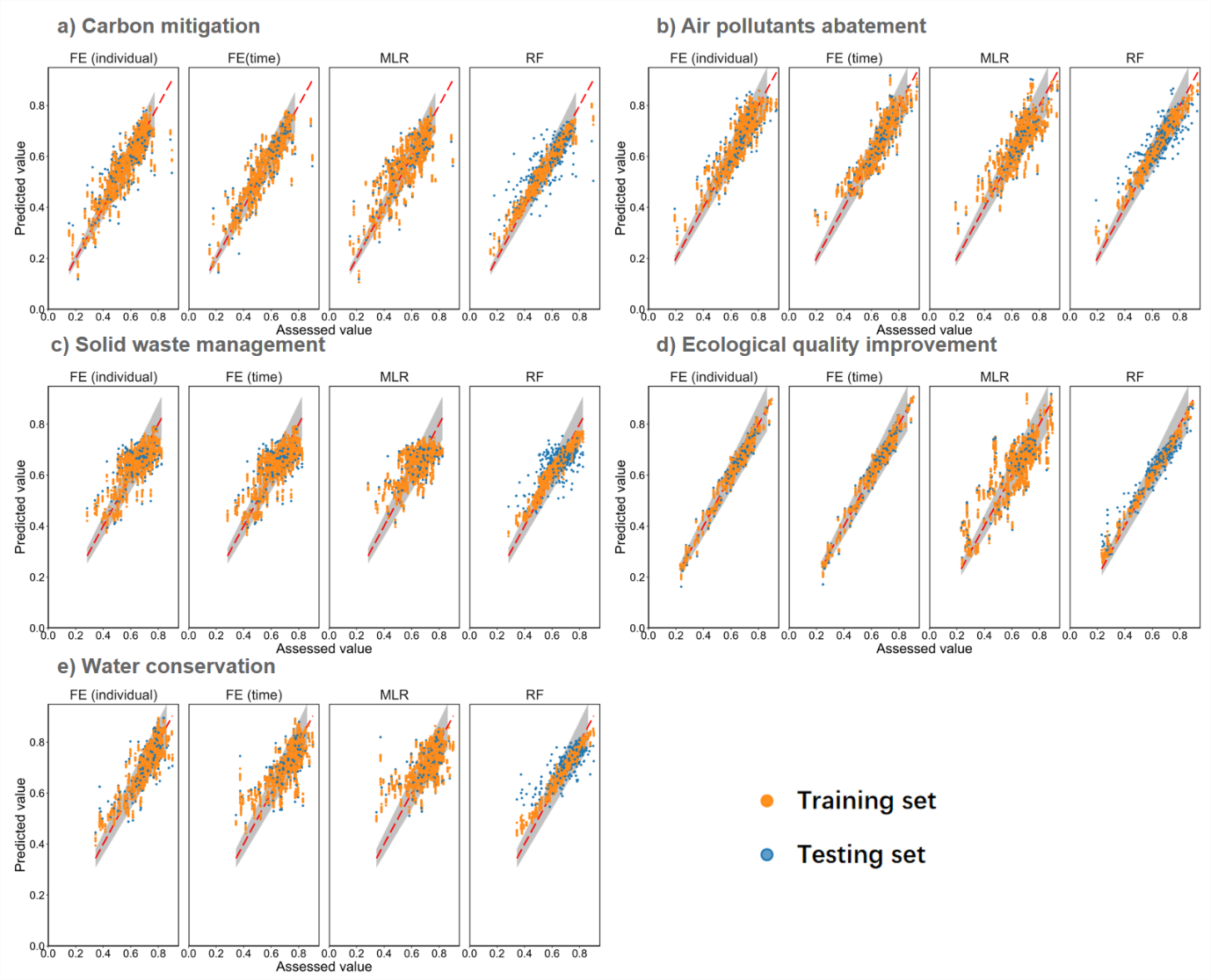


Note: The horizontal and vertical coordinates of the points on the red dashed line are equal, and the shaded areas indicate 90% confidence intervals. We find that the predicted values of the random forest model are closest to the actual values. In addition, the yellow dots represent the model prediction results for the training set, while the blue dots represent the prediction results for the test set. From the figure, we observe that the random forest model performs worse on the test set compared to the training set, indicating that the model has not been overfitted.

**Table S6.** Results of the fixed effects model analysis of the importance of socio-economic factors

| Corresponding subindustry | Carbon mitigation | Air pollutants abatement | Solid waste management | Ecological quality improvement | Water conservation |
| --- | --- | --- | --- | --- | --- |
| Processing of petroleum` | -0.459*** | -0.104 | 0.191 | -0.348*** | -0.182 |
| Manufacture of chemical products | 0.129 | -0.057 | -0.258 | 0.136 | 0.115 |
| Manufacture of chemical fibers | 1.705* | 0.360 | -0.124 | 1.386*** | -1.601* |
| Manufacture of rubber | 0.303 | -0.294 | -0.302 | 0.131 | -0.999*** |
| Manufacture of non-metallic mineral products | -0.22 | 0.061 | -0.052 | -0.340*** | 0.211 |
| Smelting of ferrous metals | -0.065 | 0.142 | 0.035 | 0.084* | 0.447*** |
| Smelting of non-ferrous metals | 0.038 | 0.097 | 0.152 | -0.087 | -0.193 |
| Manufacture of metal products | 0.093 | -0.105 | -0.264* | -0.032 | -0.351*** |
| Manufacture of electronic equipment | 0.136 | -0.281* | -0.015 | 0.109 | 0.218 |
| Recycling and disposal of waste | 0.737 | 1.533 | 1.615 | -1.078* | -0.019 |
| Construction | -0.138 | -0.252** | -0.089 | -0.027 | 0.240** |
| Transportation | -0.356 | -1.645* | -0.935 | -1.100*** | -0.460 |
| Service sector | -0.042 | 0.203 | 0.059 | -0.291*** | -0.247 |
| Energy intensity | -0.098*** | -0.030 | -0.066 | -0.025. | -0.024 |
| Urbanization level` | 0.310 | 1.219*** | 0.313 | 0.409*** | -0.022 |
| Pop | 0.135 | 0.342*** | 0.059 | -0.079 | -0.485*** |
| Expenditure for science and technology | 0.900 | 0.258 | -0.364 | -0.719* | 1.032 |
| Expenditure for environment protection | -0.594 | 0.621 | -0.469 | -0.078 | -0.665 |
| Expenditure for agriculture, forestry and water conservation | -0.104 | 0.325 | 0.225 | -0.460*** | 0.367 |
| Province FE | Yes | Yes | Yes | Yes | Yes |
| Year FE | Yes | Yes | Yes | Yes | Yes |
| Observations | 360 | 360 | 360 | 360 | 360 |
| R-squared | 0.1755 | 0.2022 | 0.0616 | 0.3292 | 0.3012 |

Note: ***, **, * are significant at 0.001, 0.01 and 0.05 levels, respectively. Robust standard errors in parentheses.

**IV. Driving factor analysis for the performance of each subsystem**

We analyzed the interaction of 19 socio-economic factors with environmental issues, as shown in Figure. S4. Although certain sectors are not key socio-economic factors, they still significantly impact many environmental issues. For example, in the solid waste management subsystem, besides the six important sectors selected, we found that the SHAP value for petroleum processing shows a significant downward trend with the growth of its output. We have also identified similar missing factors in other subsystems. These analyses reveal the non-linear relationship between socio-economic factors and environmental problems, providing theoretical support for promoting the governance of multiple environmental objectives through industrial chain restructuring in the future.

Additionally, based on the SHAP model's output values for each sample, we averaged them by province. The magnitude of these values reflects the average degree of influence various socio-economic factors have on environmental governance in the provinces during the study period. The positive or negative nature of these values indicates the direction of the influence. To more clearly identify the influence of socio-economic factors on environmental governance in each province, we standardized the averages and used hierarchical analysis to classify all the samples by province and socio-economic sector. The results further explore the influence of each socio-economic sector on environmental governance at the provincial level. For instance, while the main text reveals significant negative impacts of energy intensity on various environmental subsystems at the national level, not all regions require urgent energy efficiency improvements. Figure. S5. shows that regions highly dependent on energy production and processing, such as Inner Mongolia, Liaoning, Shanxi, and Ningxia, are constrained by inefficient energy utilization, slowing their environmental governance development. Conversely, in eastern coastal cities like Beijing, Shanghai, and Jiangsu, higher energy efficiency has not hindered the improvement of environmental governance levels.

Finally, our analyses show that different regions are not equally affected by the same socio-economic sectors, nor are they affected in the same direction. For example, certain sectors significantly impact environmental governance levels in only a few regions, which may relate to the current state of local socio-economic development. Socio-economic development levels and ecological environment statuses vary by region, and locally tailored approaches can greatly improve the effectiveness of environmental governance, reducing resource waste caused by inappropriate policies and measures. This chapter's research provides a solid theoretical basis for exploring appropriate environmental governance paths for each region.

**Figure S4.** SHAP scatter dependence plots for each subsystem


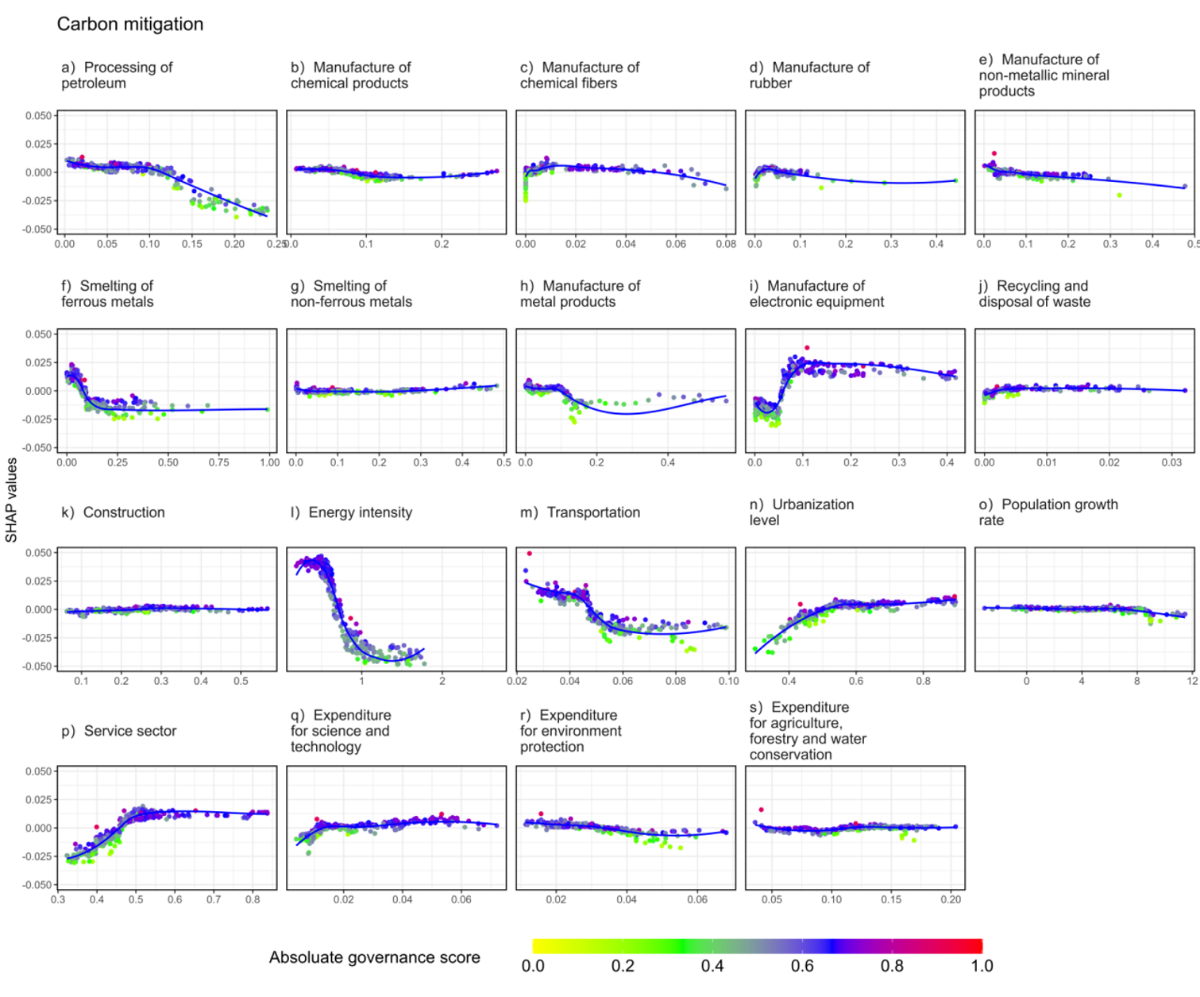


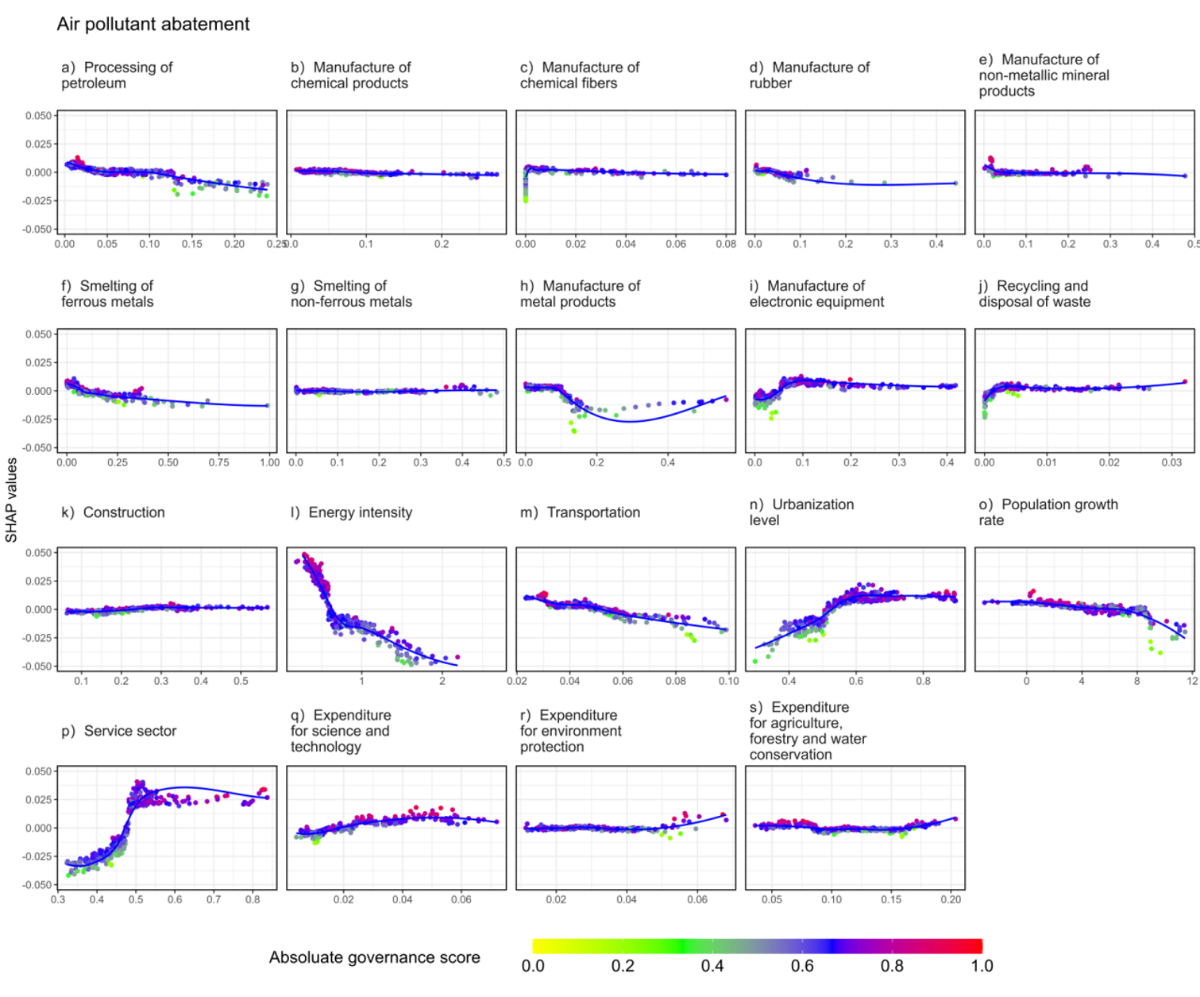


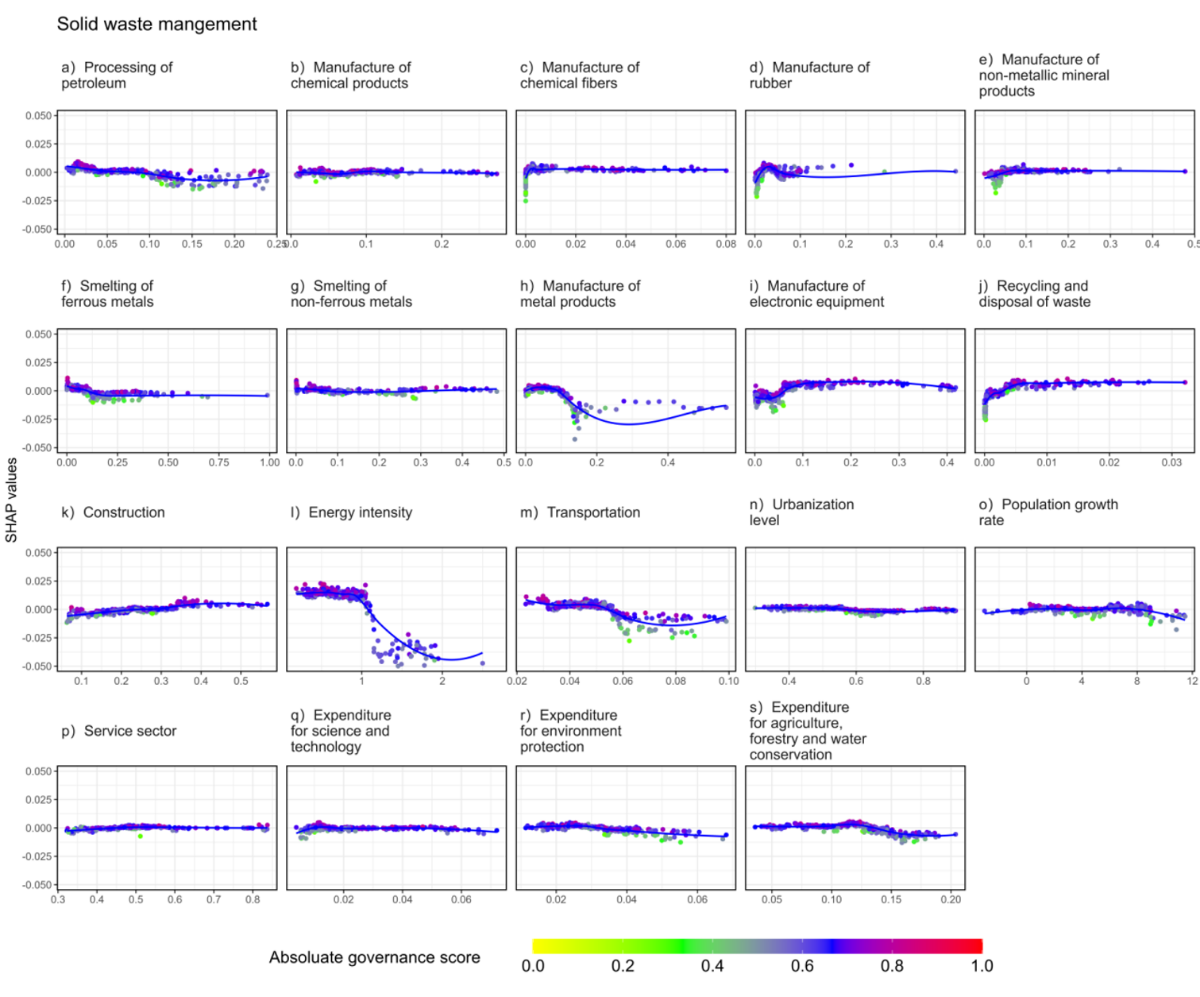


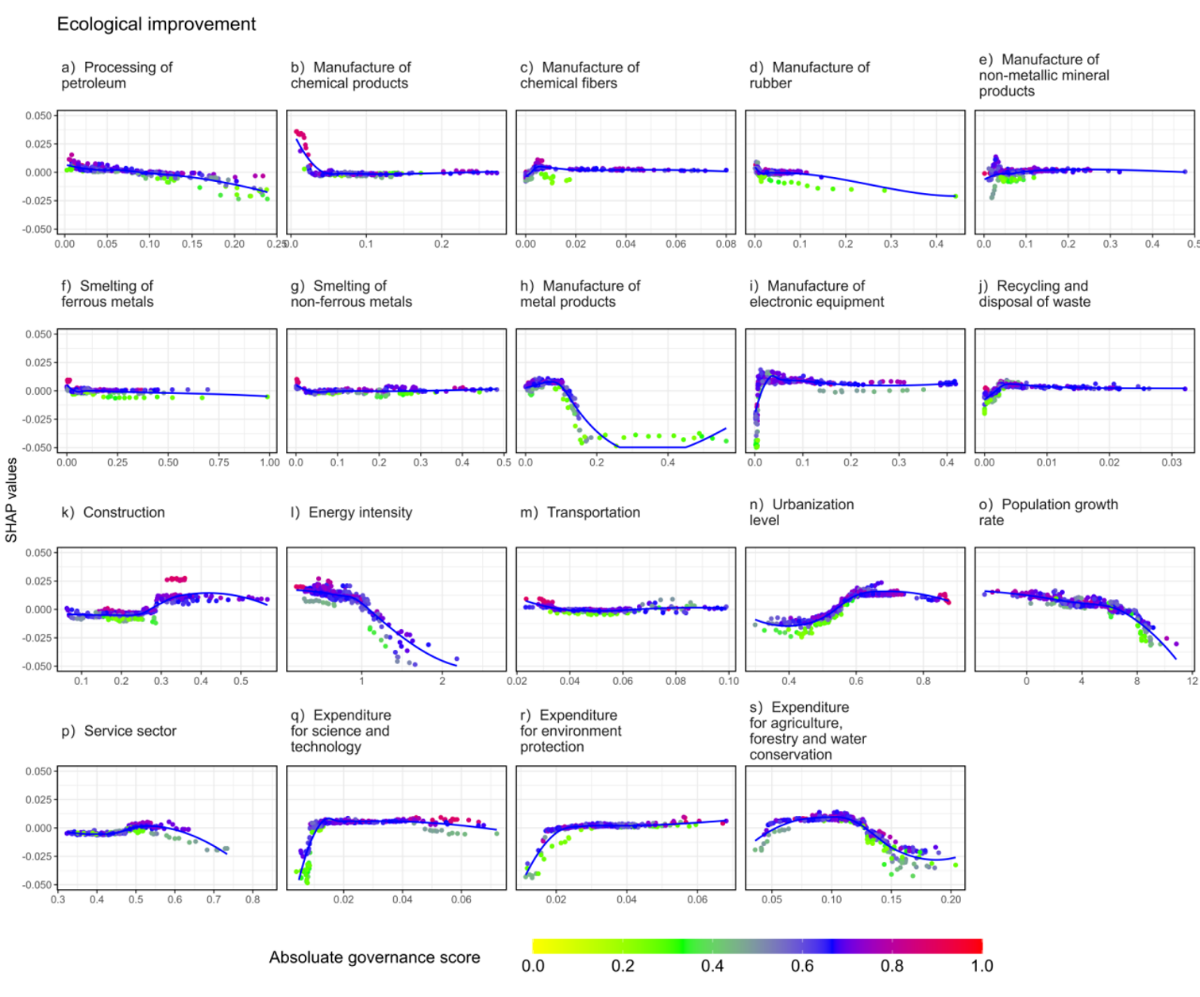


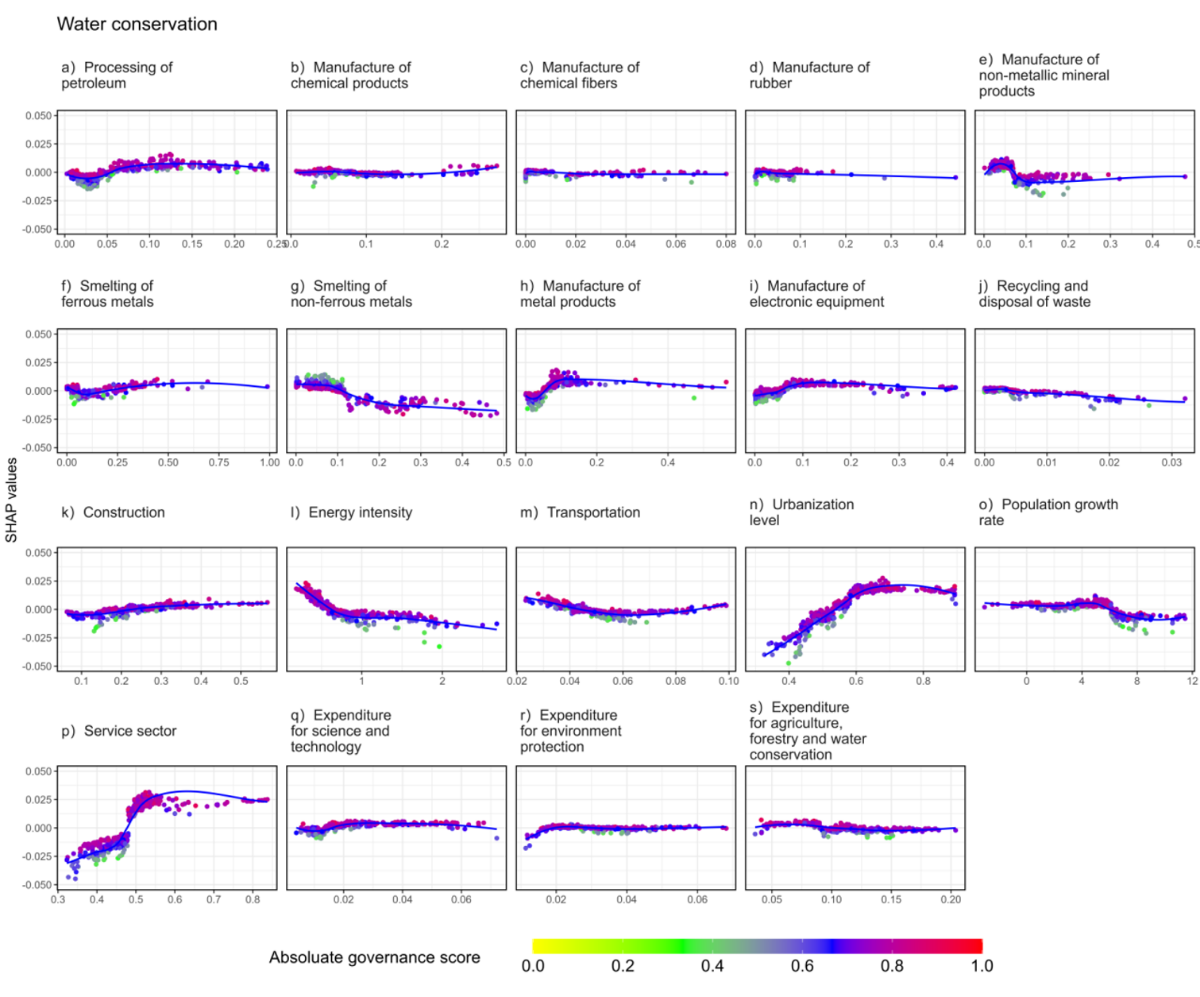


Note: (a)-(e) respectively present the interaction between all driving factors and their SHAP values in predicting the governance performance of the corresponding subsystem. A locally weighted scatterplot smoothing (LOESS) line is added to illustrate the changing trend. The color of the dots indicates the absolute governance score of a certain subsystem of the province. Analyzing the governance performance of the 19 socio-economic sectors for each subsystem effectively complements the conclusions drawn from our study. For example, in the solid waste management subsystem, except for the six important sectors selected, we find that the SHAP value of the processing of petroleum shows a significant downward trend with the growth of its output. We have also found similar missing gates in other subsystems.

**Figure S5.** Significance of socio-economic predictors for each subsystem at the provincial level


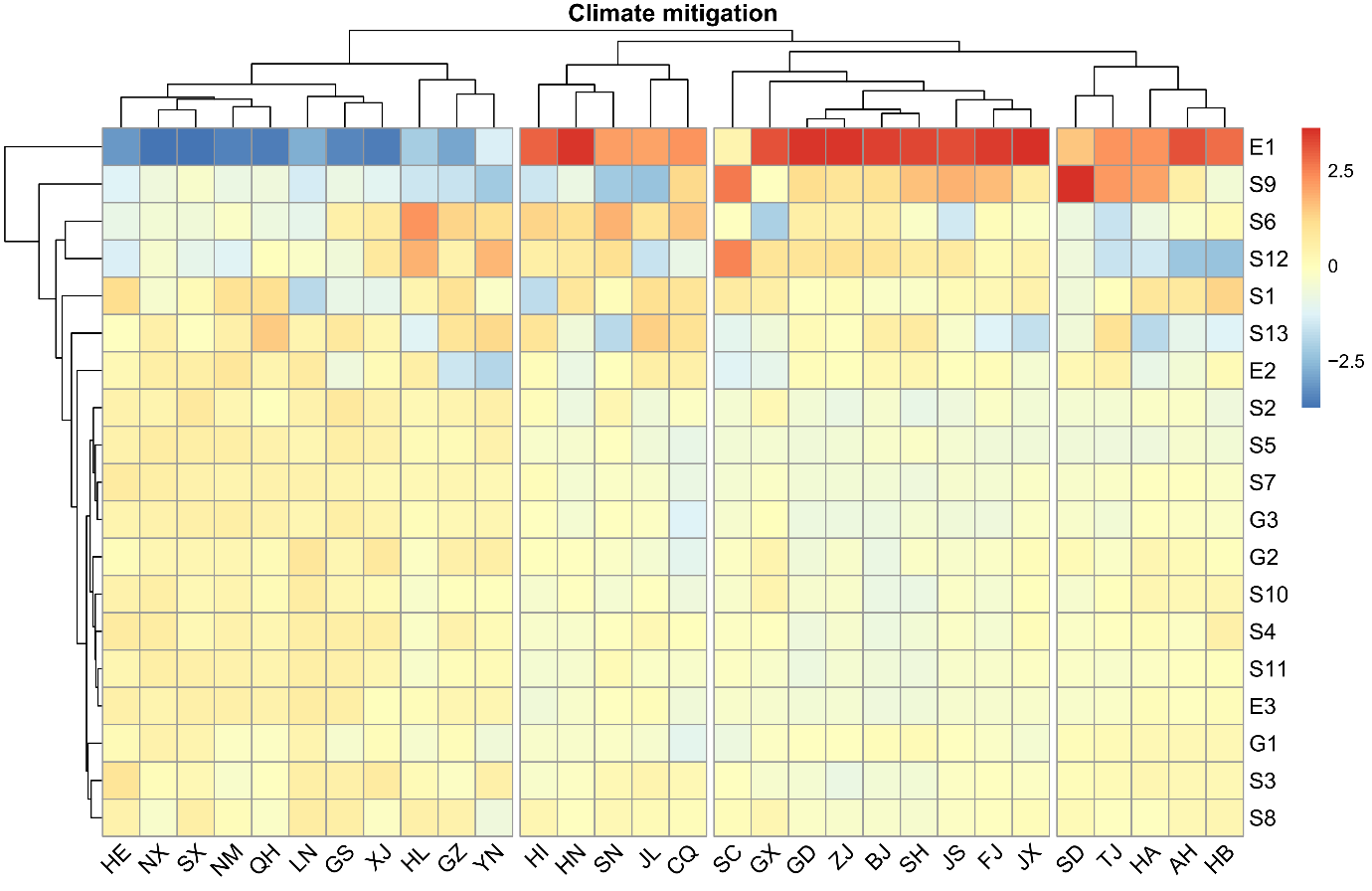


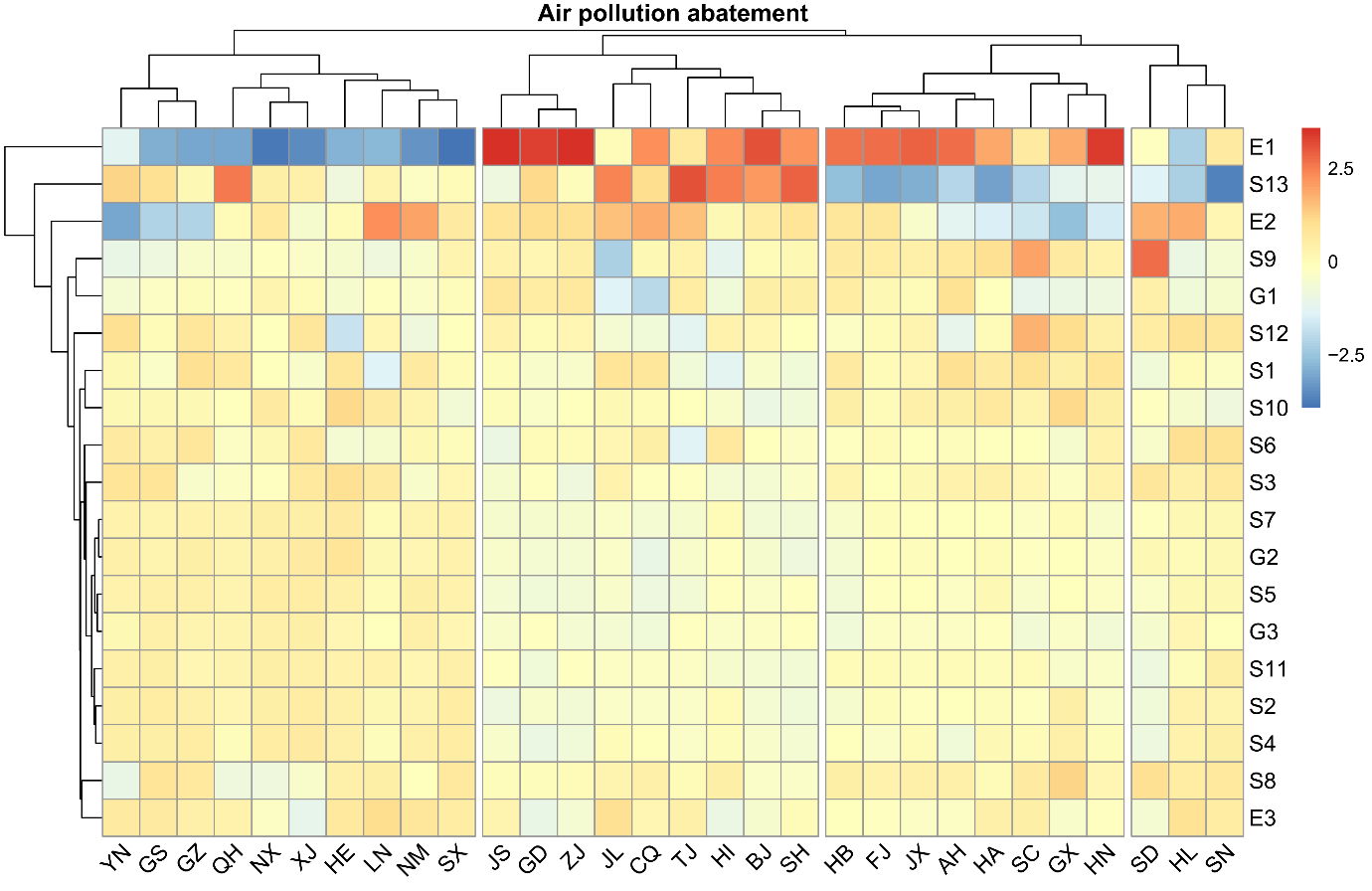


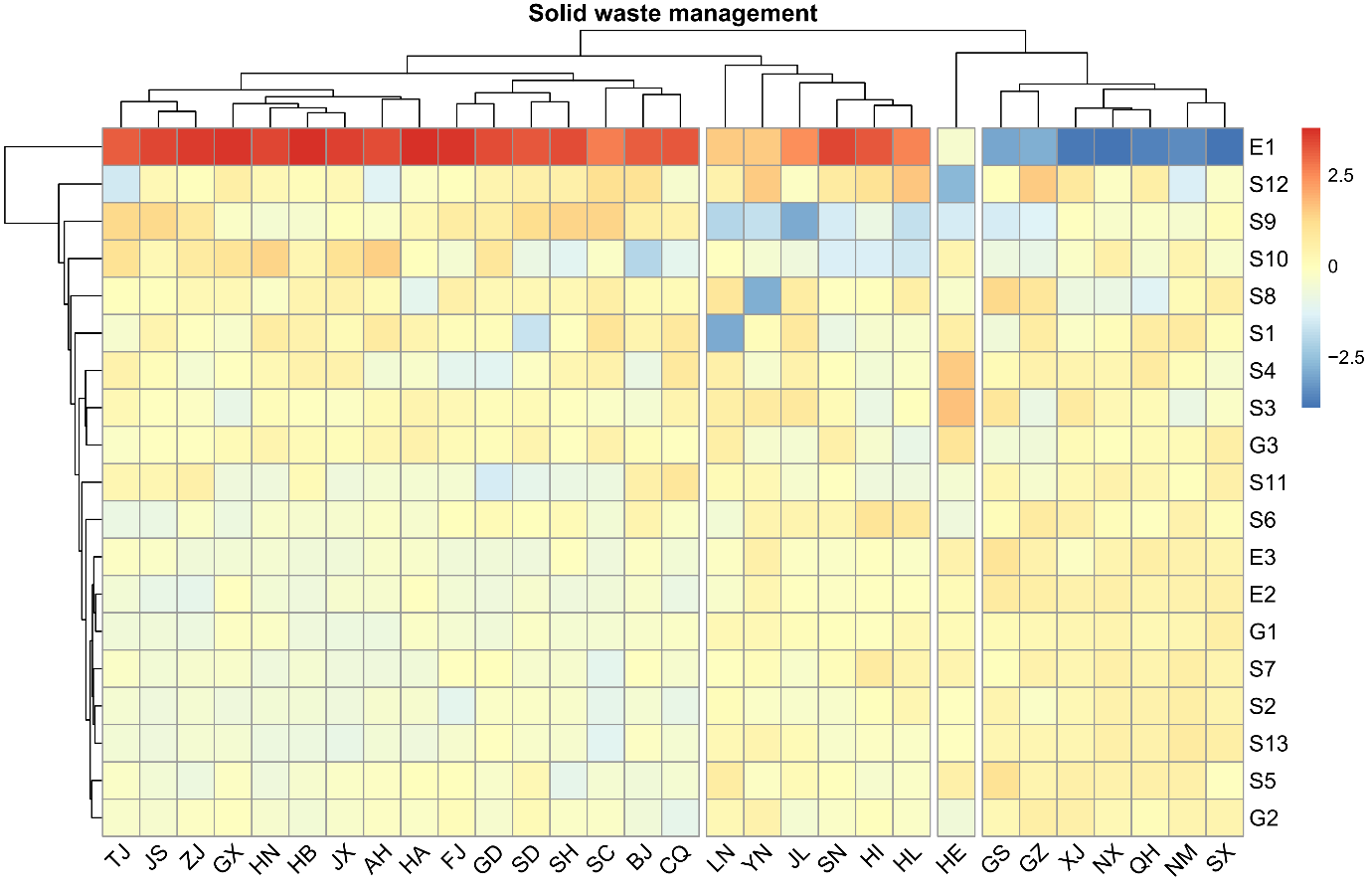


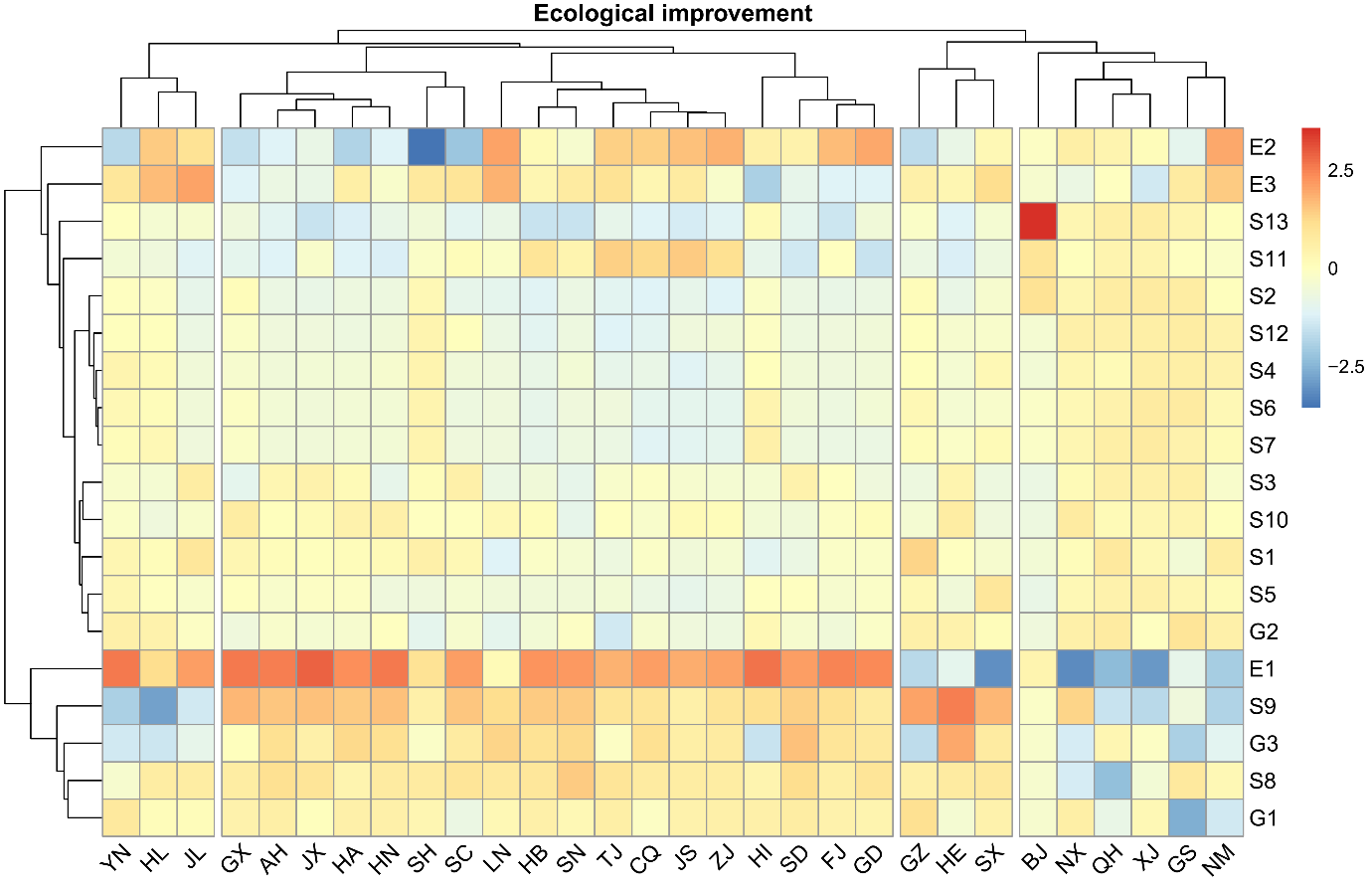


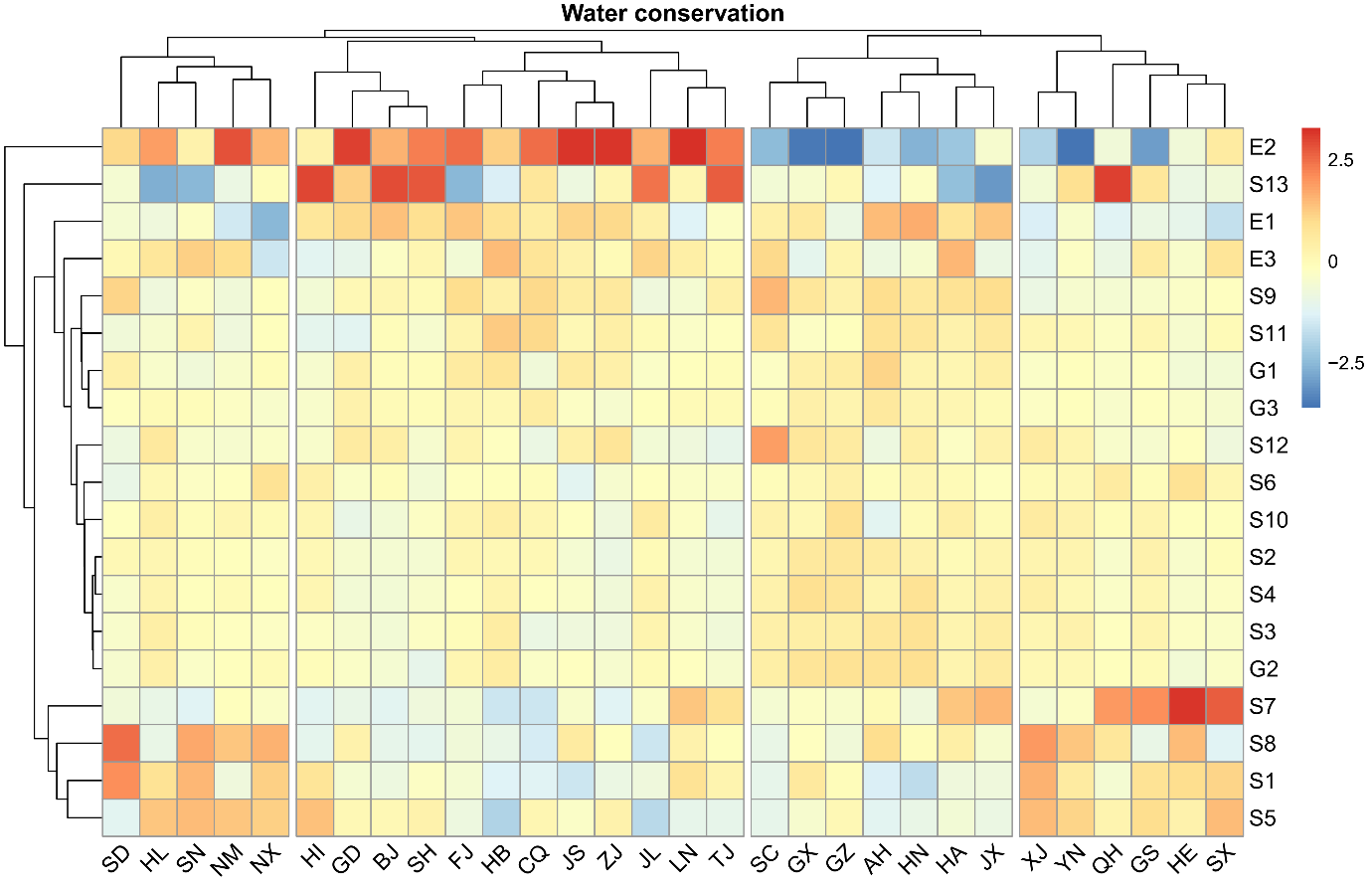


Note: We standardized the average of the SHAP values for each province and used the standardization results to classify the provinces. We did not specifically consider the number of categories when conducting the hierarchical cluster analysis, and the division into four categories is simply to show more clearly the differences in how provinces are affected by socio-economic factors (e.g., a particular socio-economic sector may only have a significant effect on a small number of provinces). The color of the boxes depends on the degree and direction of the influence of the socio-economic sector. Redder colors indicate that the development of the socio-economic sector is more favorable to environmental governance, while blue colors indicate that its development hinders the improvement of environmental governance.

**V. Provinces are categorized according to the** **synergetic governance levels**

**Figure S6.** Comparing the synergetic governance level among the different clusters


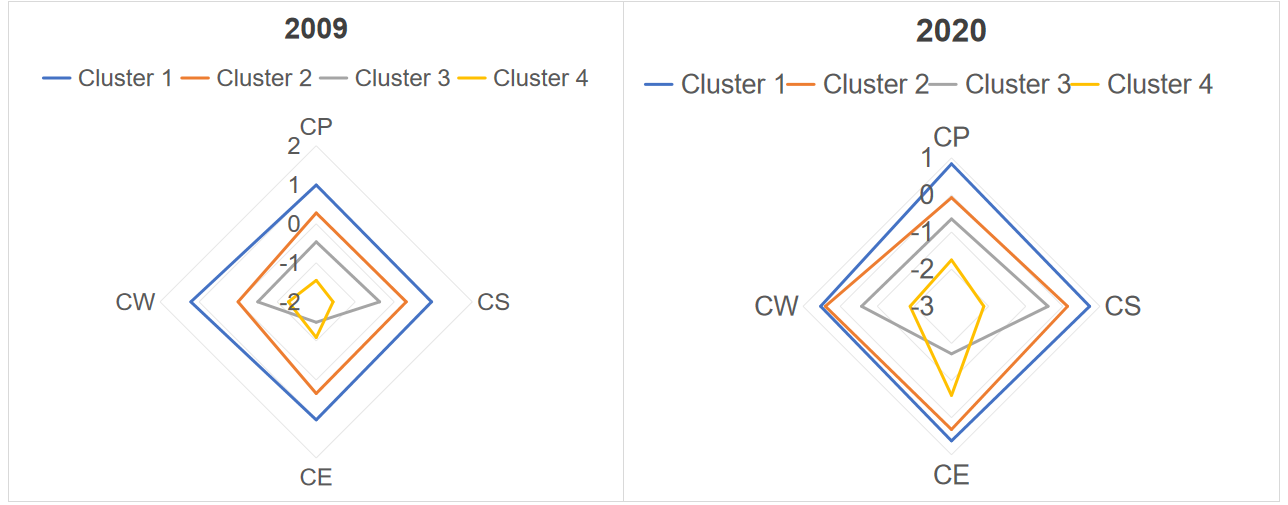


Note: In order to reflect more clearly the differences between the levels of synergistic governance in different clusters, we set the average of the synergistic governance scores of all the provinces within a cluster as the level of synergistic governance in this cluster and normalize it. Overall, the levels of synergistic governance in clusters 1-4 shows a significant decreasing trend, but the level of carbon-ecological synergistic governance in cluster 3 is significantly lower than that in cluster 4.

**Figure S7.** Results of the spatial distribution of the clusters


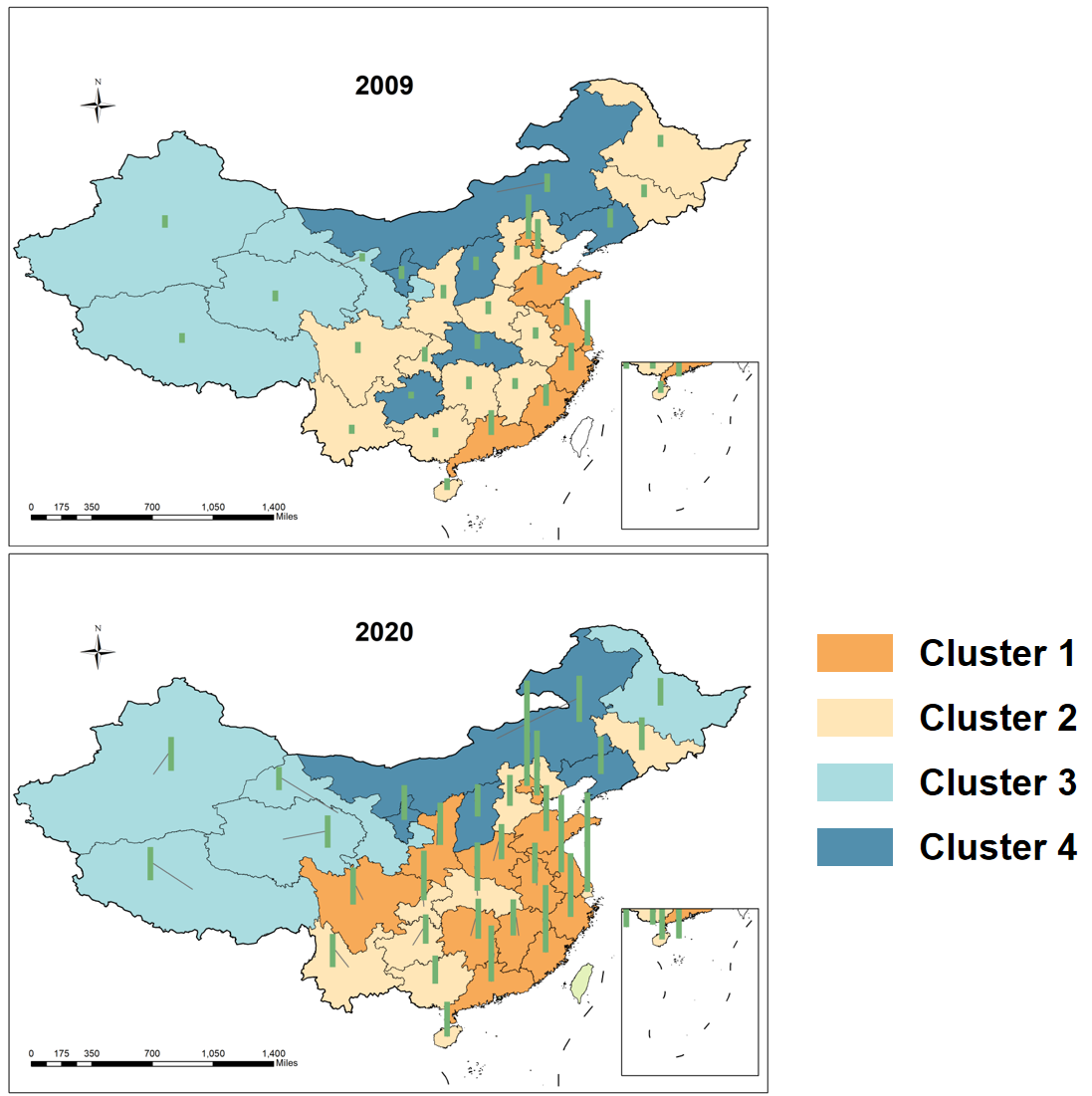


Note: Figure. S7 shows the spatial distribution of clusters in 2009 and 2020, with the height of the green bar indicating the size of the provincial GDP per capita. We find that cluster 1 is mainly distributed in the eastern coastal areas, cluster 3 is mainly distributed in the northwest and Qinghai-Tibet Plateau, and cluster 4 is mainly distributed in the areas rich in mineral resources in the north and northeast.

**VI. Impact of environmental policy implementation on synergistic governance**

**Table S7.** The impact of air quality policy on the synergetic governance

|  | Air pollutants abatement | | Carbon mitigation | | Carbon mitigation & Air pollutants abatement | |
| --- | --- | --- | --- | --- | --- | --- |
| air quality policy | 0.1434*** | 0.0613*** | 0.1329*** | 0.1198*** | 0.1081*** | 0.0796*** |
|  | (0.0074) | (0.0103) | (0.0066) | (0.0106) | (0.0039) | (0.0058) |
| energy consumption |  | -0.0011*** |  | -0.0006* |  | -0.0006*** |
|  |  | (0.0002) |  | (0.0003) |  | (0.0001) |
| industrial structure |  | 0.5884*** |  | -0.048 |  | 0.1531* |
|  |  | (0.1119) |  | (0.1154) |  | (0.0636) |
| perGDP |  | 0.005 |  | 0.0018 |  | 0.0014 |
|  |  | (0.0036) |  | (0.0037) |  | (0.0021) |
| lnpop |  | 0.1719 |  | 0.0064 |  | 0.0412 |
|  |  | (0.1186) |  | (0.1222) |  | (0.0674) |
| technology level |  | 0.4304 |  | 0.4487 |  | 0.2935 |
|  |  | (0.6695) |  | (0.6903) |  | (0.3806) |
| Province FE | Yes | Yes | Yes | Yes | Yes | Yes |
| Year FE | Yes | Yes | Yes | Yes | Yes | Yes |
| Observations | 360 | 360 | 360 | 360 | 360 | 360 |
| R-squared | 0.5274 | 0.6609 | 0.6736 | 0.6832 | 0.6968 | 0.7450 |

Note: ***, **, * are significant at 0.001, 0.01 and 0.05 levels, respectively. Robust standard errors in parentheses.

**Table S8.** The impact of water conservation policy on synergetic governance

|  | Water conservation | | Carbon mitigation | | Carbon mitigation & Water conservation | |
| --- | --- | --- | --- | --- | --- | --- |
| water conservation policy | 0.0846*** | 0.0262* | 0.1253** | 0.0482** | 0.0727*** | 0.0265** |
|  | (0.0069) | (0.0127) | (0.0085) | (0.0161) | (0.0045) | (0.0084) |
| energy consumption |  | -0.0056* |  | -0.0068* |  | -0.0051* |
|  |  | (0.0026) |  | (0.0033) |  | (0.0018) |
| industrial structure |  | 0.1367 |  | 0.318* |  | 0.1589* |
|  |  | (0.1482) |  | (0.1803) |  | (0.0941) |
| perGDP |  | 0.0204*** |  | 0.0153** |  | 0.0115*** |
|  |  | (0.0037) |  | (0.0046) |  | (0.0024) |
| lnpop |  | -0.4146** |  | 0.1259 |  | -0.1041 |
|  |  | (0.1253) |  | (0.1569) |  | (0.0818) |
| technology level |  | 1.5553* |  | 0.3564 |  | 0.5695 |
|  |  | (0.7074) |  | (0.8865) |  | (0.4625) |
| Province FE | Yes | Yes | Yes | Yes | Yes | Yes |
| Year FE | Yes | Yes | Yes | Yes | Yes | Yes |
| Observations | 360 | 360 | 360 | 360 | 360 | 360 |
| R-squared | 0.3125 | 0.5382 | 0.3981 | 0.4782 | 0.4362 | 0.6779 |

Note: ***, **, * are significant at 0.001, 0.01 and 0.05 levels, respectively. Robust standard errors in parentheses.

**Table S9.** The impact of carbon mitigation policy on synergetic governance

|  | Carbon mitigation | | Air pollutants abatement | | Water conservation | | Carbon mitigation & Air pollutants abatement | | Carbon mitigation & Water conservation | |
| --- | --- | --- | --- | --- | --- | --- | --- | --- | --- | --- |
| carbon mitigation policy | 0.1022*** | 0.0959* | 0.1484*** | 0.1362* | 0.0996* | 0.0217 | 0.0803*** | 0.0609* | 0.0786*** | 0.0081 |
|  | (0.0125) | (0.0228) | (0.0215) | (0.0278) | (0.0173) | (0.0181) | (0.0139) | (0.0062) | (0.0125) | (0.0119) |
| energy consumption |  | -0.0007* |  | -0.0009*** |  | -0.0006* |  | -0.0006** |  | -0.0005** |
|  |  | (0.0003) |  | (0.0003) |  | (0.0002) |  | (0.0001) |  | (0.0002) |
| industrial structure |  | 0.6816*** |  | 0.8649*** |  | 0.3344** |  | 0.5156*** |  | 0.3588*** |
|  |  | (0.1361) |  | (0.1062) |  | (0.1076) |  | (0.0723) |  | (0.0711) |
| perGDP |  | 0.0171*** |  | 0.0143*** |  | 0.0203*** |  | 0.0106*** |  | 0.0126*** |
|  |  | (0.0049) |  | (0.0039) |  | (0.0039) |  | (0.0026) |  | (0.0026) |
| lnpop |  | 0.1364 |  | 0.3175* |  | -0.4391*** |  | 0.1485 |  | -0.0969 |
|  |  | (0.1665) |  | (0.1299) |  | (0.1317) |  | (0.0883) |  | (0.0869) |
| technology level |  | 0.2406 |  | 0.3709 |  | 1.4859* |  | 0.1987 |  | 0.5061 |
|  |  | (0.8974) |  | (0.0143) |  | (0.7098) |  | (0.4759) |  | (0.4687 |
| Province FE | Yes | Yes | Yes | Yes | Yes | Yes | Yes | Yes | Yes | Yes |
| Year FE | Yes | Yes | Yes | Yes | Yes | Yes | Yes | Yes | Yes | Yes |
| Observations | 360 | 360 | 360 | 360 | 360 | 360 | 360 | 360 | 360 | 360 |
| R-squared | 0.2077 | 0.6644 | 0.2712 | 0.6288 | 0.0911 | 0.4228 | 0.0911 | 0.6012 | 0.1072 | 0.5245 |

Note: ***, **, * are significant at 0.001, 0.01 and 0.05 levels, respectively. Robust standard errors in parentheses.

**VII. Methodology for the construction of a higher dimensional synergistic governance model**

In the main text, we only considered the synergetic governance of two systems and focused on interactions between the climate change and one other environmental system. However, modeling without considering higher dimensions of synergetic governance may overlook complex interactions between environmental elements (3). For instance, certain industries, like those involving clean energy sources such as wind, solar, and lithium-ion batteries, can significantly enhance the synergetic control of carbon emissions and air pollution. However, the installation of these technologies may alter land use patterns, potentially causing ecological damage (4). Inadequate handling of waste generated by clean energy applications can lead to solid waste and water pollution (5, 6). Therefore, focusing solely on the synergistic treatment of two aspects may disregard potential synergies or antagonistic effects on other environmental issues.

Therefore, we included a discussion on the Coupling Coordination Degree (CCD) model in the appendix, illustrating how to construct a higher-dimensional collaborative governance model. The discussion also outlines calculation methods to represent the interactions among multiple environmental governance processes (coupling degree), the overall governance level (development degree) and the collaborative governance level (coupling coordination degree).

First, we should construct an indicator framework containing multidimensional environmental governance objectives and collect data for each indicator. Then, the collected data will be used to calculate the absolute governance score of each subsystem through equations (1)-(5). Finally, the absolute governance score of each subsystem is input into the coupling coordination degree model, and the coupling degree, development degree and coupling coordination degree between multiple subsystems are calculated through equations (6)-(8). Equations (1)-(5) can be found in the main text and equations (6)-(8) are shown below:

$$\begin{aligned} C=\frac{n\times{(u_{1}\times u_{2}\times\cdots\times u_{n})}^{1/n}}{u_{1}+u_{2}+\cdots+u_{n}}\#(6)\# \end{aligned}$$

where *C* is the coupling degree, indicating the extent of synergistic changes, or the degree of interdependence for the many subsystems in addressing environmental issues. $u_{1}$, $u_{2}$,…, $u_{n}$ are the absolute governance levels of the n environmental subsystems, respectively. Next, we specify:

$$\begin{aligned} \begin{aligned} T=\alpha_{1}\times u_{1}+\alpha_{2}\times u_{2}+\cdots+\alpha_{n}\times u_{n}\#(7)\# \end{aligned}\# \end{aligned}$$

$T$ is the development degree, indicating the overall progress of absolute governance level for the many subsystems, which can reflect the quality of synergy. Then, $\alpha_{1}$, $\alpha_{1}$,… $\alpha_{n}$ are the coefficients of weights to be referenced for the n environmental subsystems, respectively. In related studies we were able to take principal component analysis, grey correlation or entropy weights to determine the value of *α*. In general, we usually assumed that each subsystem is equally important to evaluate the development degree, so $\alpha_{1}$ *=*$\alpha_{2}$ *=…=*$\alpha_{n}$*=*1/n*.*

$$\begin{aligned} CCD=\sqrt{C\times T}\#(8)\# \end{aligned}$$

Finally, we calculated *CCD* scores to reflect the synergetic governance level for all environmental subsystems. The higher the CCD values, the better performance of synergy achieved by the all environmental subsystems.

In addition, we are able to change the number of environmental subsystems in the input coupling coordination degree model to explore the synergistic governance level of different combinations of subsystems, thus increasing the flexibility of the model.

**VIII. Analysis of the synergistic effect and obstacle degree between climate action and comprehensive environmental governance**

In order to explore the synergy between climate action and comprehensive environmental governance in China, based on the content of the previous section, a five-dimensional coupled coordination degree model was constructed to calculate the five-element synergetic governance scores of carbon mitigation in each province and other four environmental subsystems. The obstacle degree model is used to diagnose the main environmental problems that restrict the improvement of the level of synergetic governance in different provinces. The specific equation is as follows:

$$\begin{aligned} O_{j}=\frac{F_{j}I_{j}}{\sum_{j=1}^{m} F_{j}I_{j}}\#\left( 9 \right) \end{aligned}$$

Where $F_{j}$ represents the weight of the environment subsystem *j* in the whole synergistic governance system, and the weight coefficient of the five environment subsystems in this study is 1/5. $I_{j}$ represents the degree of deviation between the standardized value of the environmental subsystem *j* absolute governance score and the optimal value 1, which is equal to 1 minus its standardized value. *m* is the total number of subsystems in the synergistic governance system. $O_{j}$ is the obstacle of the environmental subsystem *j* to the improvement of the level of synergistic governance, and the greater the degree of obstacle, the greater the restriction of the environmental subsystem on regional synergistic governance.

The results of the study are shown in Fig. S8 and Fig. S9.

**Figure S8.** The five-element synergetic governance scores of carbon mitigation in each province and other four environmental subsystems


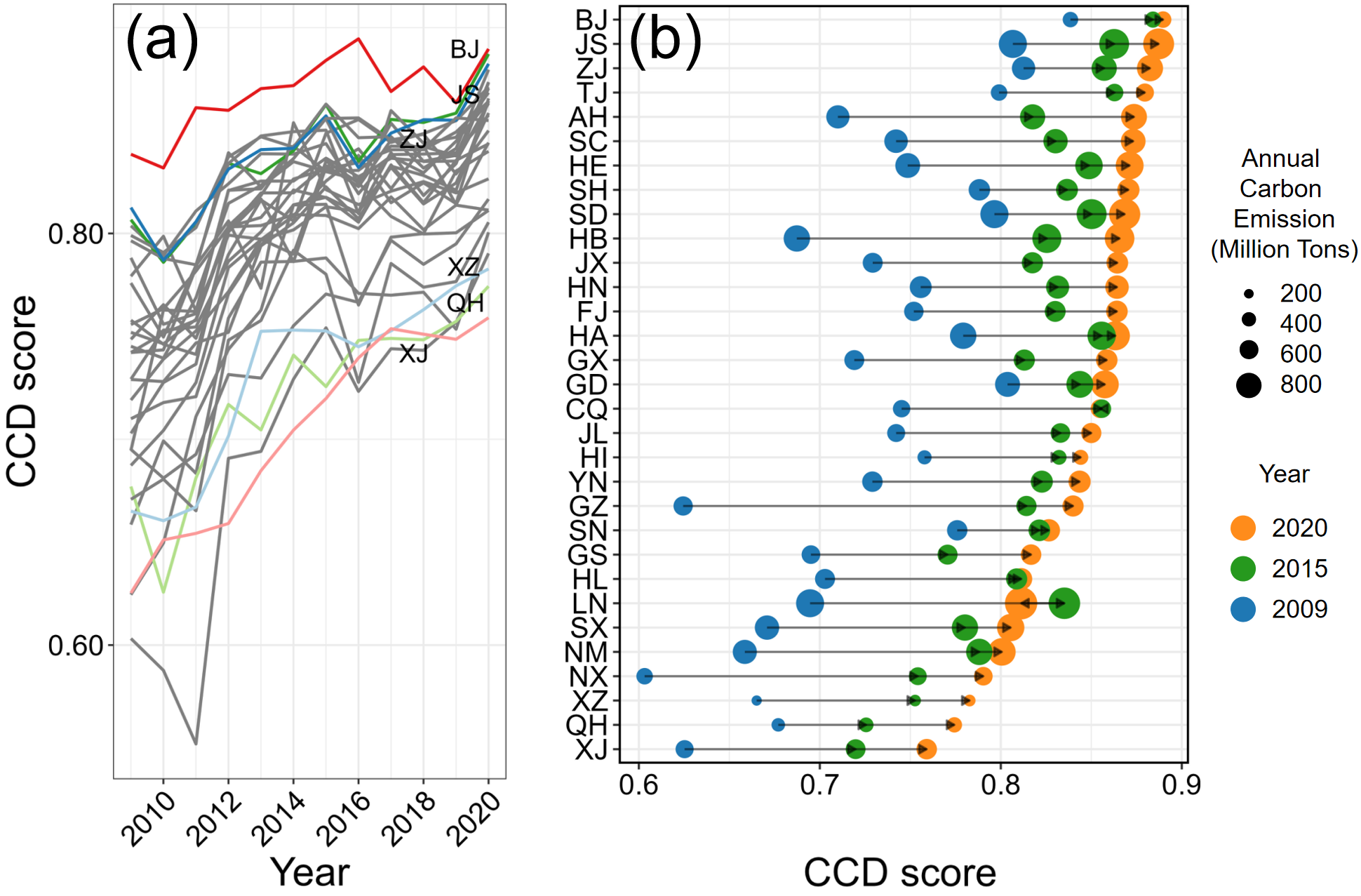


Note: Fig.S8A shows the interannual variation of the synergetic governance scores of the five subsystems in each province. From 2009 to 2020, the level of synergetic governance of multi-dimensional environmental problems in various provinces has been significantly improved, showing a rapid growth trend from 2009 to 2015, and then the growth rate has slowed down significantly. During the period of the study, the level of synergetic governance in many provinces showed some fluctuations, which may be related to the fluctuation of the absolute governance scores of their carbon mitigation subsystem and solid waste management subsystem. Fig.S8B shows the synergetic governance scores of the five subsystems of each province in 2009, 2015 and 2020. After 11 years of governance process, climate action and comprehensive environmental governance in most provinces of China have formed a strong synergistic effect (CCD>0.8), but there are still some provinces in the western region where multi-dimensional environmental problems have not been well coupled. From the perspective of specific provinces, developed coastal provinces such as Beijing (BJ), Jiangsu (JS) and Zhejiang (ZJ) have a high level of synergetic governance of multi-dimensional environmental problems, which effectively coordinate environmental protection on the basis of ensuring high-level economic development. However, due to the restriction of natural endowment and production level, the sustainable development of western provinces such as Xinjiang (XJ), Qinghai (QH) and Tibet (XZ) is still facing the challenge of environmental problems such as high-intensity pollutant discharge and ecological destruction.

**Figure S9.** The results of the obstacle degree analysis of the subsystem


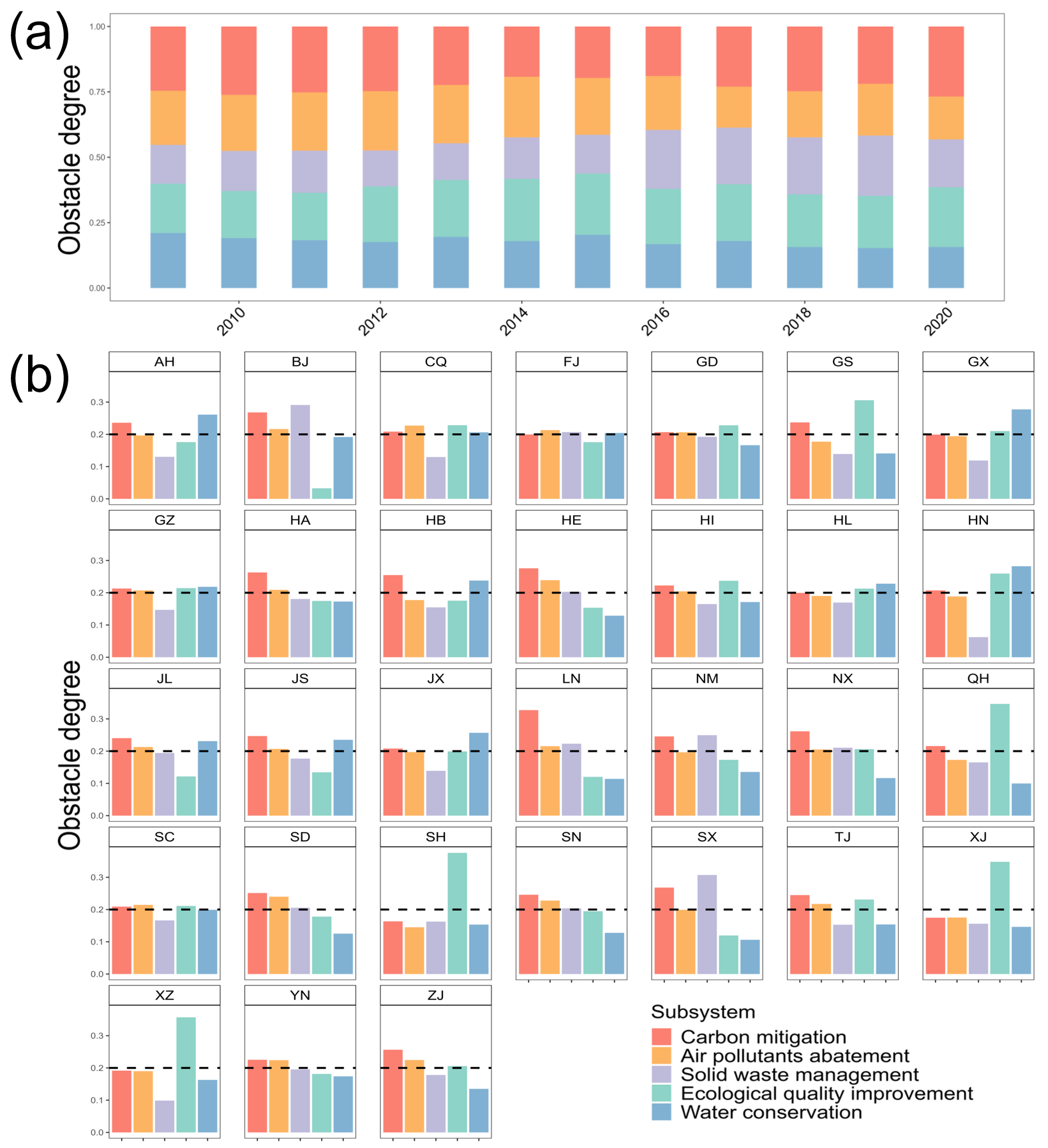


Note: Fig.S9A shows the interannual variation of obstacles at the national level. From 2009 to 2020, the obstacles of air pollutants abatement and water resources conservation subsystems decreased significantly, while the constraints of carbon mitigation and ecological quality improvement subsystems on synergetic governance have been strengthened, which has become the main obstacle to the improvement of synergetic governance. In addition, the obstacle degree of solid waste management subsystem shows a fluctuating trend, which is consistent with the change law of its absolute governance scores. Fig.S9B reflects the distribution of obstacles in each province. The obstacle degree distribution of each subsystem in most provinces is more balanced (the obstacle degree of the five subsystems is all around 0.2), which shows that its development process is faced with a variety of environmental challenges, not a single problem dominates its development constraints. However, the synergetic governance of some provinces is restricted by some major environmental problems. For example, carbon emission reduction subsystem and solid waste management subsystem play a significant role in Liaoning (LN), Inner Mongolia (NM) and Shanxi (SX), while Xinjiang (XJ), Qinghai (QH) and Tibet (XZ) provinces are facing severe challenges of ecological problems.

**References**

1. Ambroise C & McLachlan GJ (2002) Selection bias in gene extraction on the basis of microarray gene-expression data. *Proceedings of the National Academy of Sciences of the United States of America* 99(10):6562-6566.

2. Chicco D, Warrens MJ, & Jurman G (2021) The coefficient of determination R-squared is more informative than SMAPE, MAE, MAPE, MSE and RMSE in regression analysis evaluation. *PeerJ. Computer science* 7:e623.

3. Fabian PS, Kwon H-H, Vithanage M, & Lee J-H (2023) Modeling, challenges, and strategies for understanding impacts of climate extremes (droughts and floods) on water quality in Asia: A review. *Environmental Research* 225:115617.

4. Hassan Q, Algburi S, Sameen AZ, Salman HM, & Jaszczur M (2023) A review of hybrid renewable energy systems: Solar and wind-powered solutions: Challenges, opportunities, and policy implications. *Results in Engineering* 20:101621.

5. Abdel-Shafy HI & Mansour MSM (2018) Solid waste issue: Sources, composition, disposal, recycling, and valorization. *Egyptian Journal of Petroleum* 27(4):1275-1290.

6. Traven L (2023) Sustainable energy generation from municipal solid waste: A brief overview of existing technologies. *Case Studies in Chemical and Environmental Engineering* 8:100491.
